# Human intestinal bitter taste receptors regulate innate immune responses and metabolic regulators in obesity

**DOI:** 10.1172/JCI144828

**Published:** 2022-02-01

**Authors:** Kathrin I. Liszt, Qiaoling Wang, Mona Farhadipour, Anneleen Segers, Theo Thijs, Linda Nys, Ellen Deleus, Bart Van der Schueren, Christopher Gerner, Benjamin Neuditschko, Laurens J. Ceulemans, Matthias Lannoo, Jan Tack, Inge Depoortere

**Affiliations:** 1 Gut Peptide Research Lab, Targid, University of Leuven, Leuven, Belgium.; 2 Department of Abdominal Surgery and; 3 Department of Endocrinology, University Hospitals Leuven, Leuven, Belgium.; 4 Department of Analytical Chemistry and; 5 Department of Inorganic Chemistry, Faculty of Chemistry, University of Vienna, Vienna, Austria.; 6 Leuven Intestinal Failure and Transplantation (LIFT) Center, University Hospitals Leuven, Leuven, Belgium.; 7 Gastrointestinal Motility and Sensitivity Research Group, Targid, University of Leuven, Leuven, Belgium.

**Keywords:** Gastroenterology, Metabolism, G protein&ndash;coupled receptors, Innate immunity, Obesity

## Abstract

Bitter taste receptors (taste 2 receptors, TAS2Rs) serve as warning sensors in the lingual system against the ingestion of potentially poisonous food. Here, we investigated the functional role of TAS2Rs in the human gut and focused on their potential to trigger an additional host defense pathway in the intestine. Human jejunal crypts, especially those from individuals with obesity, responded to bitter agonists by inducing the release of antimicrobial peptides (α-defensin 5 and regenerating islet–derived protein 3 α [REG3A]) but also regulated the expression of other innate immune factors (mucins, chemokines) that affected *E*. *coli* growth. We found that the effect of aloin on *E*. *coli* growth and on the release of the mucus glycoprotein CLCA1, identified via proteomics, was affected by TAS2R43 deletion polymorphisms and thus confirmed a role for TAS2R43. RNA-Seq revealed that denatonium benzoate induced an NRF2-mediated nutrient stress response and an unfolded protein response that increased the expression of the mitokine *GDF15* but also *ADM2* and *LDLR*, genes that are involved in anorectic signaling and lipid homeostasis. In conclusion, TAS2Rs in the intestine constitute a promising target for treating diseases that involve disturbances in the innate immune system and body weight control. TAS2R polymorphisms may be valuable genetic markers to predict therapeutic responses.

## Graphical abstract

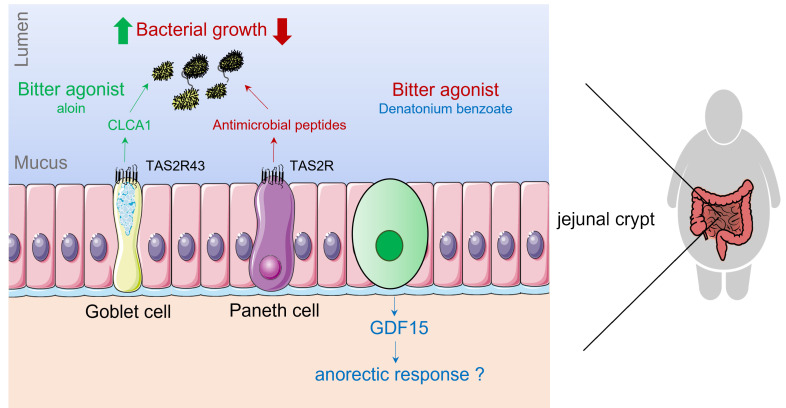

## Introduction

Bitter is an aversive taste perceived on the tongue that acts as the first defense mechanism to protect our body against the ingestion of potentially poisonous compounds (1). Bitter taste is detected by GPCRs of the taste 2 receptor family (referred to herein as TAS2Rs for humans and Tas2rs for mice), of which approximately 25 subtypes have been found in humans (2, 3).

The finding that TAS2Rs are expressed in multiple organs outside the oral cavity has prompted studies on their physiological function (4, 5). In particular, the gastrointestinal tract, which forms a critical interface between the exterior and the human body, continuously monitors the presence of possible toxic bitter compounds derived from ingested food, drinks, pharmaceuticals, or bacterial sources. The expression of TAS2Rs and components of the chemosensory signaling pathways has been described in several gut epithelial cells such as tuft cells, enteroendocrine cells (EECs), goblet cells, and Paneth cells (4, 6). So far, only for EECs and tuft cells, a physiological function of TAS2Rs has been demonstrated. For instance, TAS2Rs expressed on EECs are involved in regulating the secretion of both orexigenic (ghrelin) and anorexigenic peptides (GLP-1, PYY, CCK) that affect hunger scores and food intake in humans and mice (6–9). In mice, tuft cells orchestrate parasite type 2 immunity during helminth infection, which involves gustatory signaling pathways and Tas2rs (10). The expression of Tas2r131 has been discovered in colonic goblet cells (11) and Paneth cells (12) using a knockin mouse strain, but a functional role for TAS2Rs in the secretion of mucins or antimicrobial peptides, respectively, has not, to our knowledge, been described. Of note, in solitary chemosensory cells from human upper airways, activation of TAS2Rs by the bitter compound denatonium benzoate (DB) stimulated the secretion of antimicrobial β-defensins, which resulted in killing of the bacteria *Pseudomonas aeruginosa* (13). We therefore hypothesized that intestinal TAS2Rs may fulfill a similar role in protecting the gut against bacterial pathogens through stimulation of antimicrobial peptide secretion.

Obesity is associated with a reduction in taste-related genes in human taste buds (14), which may affect food preference and energy intake. Our previous studies showed that TAS2R expression in the gut epithelium was altered by obesity in a region-dependent manner (7). In addition, the sensitivity of ghrelin-expressing EECs to DB was impaired in the small intestine of patients with obesity (7). Similarly, it is likely that the expression and sensitivity of TAS2Rs in other cell types, such as Paneth cells and goblet cells, are affected by obesity. Altered sensing of bitter compounds in combination with reduced antimicrobial peptide levels observed in Paneth cells (15) from obese individuals may contribute to gut microbiota dysbiosis (16).

The aim of this study was to further understand the role of TAS2Rs in human gut physiology, with a special focus on the activation of Paneth and goblet cells during health and obesity. More specifically, we investigated (a) the acute effect of bitter agonists on the activation of innate immune factors (defensins, mucins) in primary jejunal crypts from lean and/or obese individuals; (b) the functional effect of the release of these molecules on *E*. *coli* growth, with a focus on the effect of DB, a broadly tuned bitter agonist (8 different TAS2Rs), and aloin, a TAS2R43- and TAS2R31-specific agonist; (c) the role of TAS2R43 in the observed effects by taking advantage of the existence of a whole gene deletion and single nucleotide polymorphism for TAS2R43 in humans (17, 18). We performed transcriptomics analysis to gain a broader understanding of which pathways are regulated by bitter compounds.

To our knowledge, this is the first study to provide an in-depth analysis of the effect of bitter compounds in extra-oral tissue at the molecular level in humans. Our study shows that stimulation of jejunal crypts, especially those from individuals with obesity, with bitter compounds induced the release of antimicrobial peptides and proteins that determine the structural arrangement of the mucus (CLCA1) to affect *E*. *coli* growth. Prolonged incubation with bitter compounds affected the mRNA expression of immune factors (defensins, mucins, chemokines). In addition, we uncovered pathways that could explain how bitter compounds affect food intake and triglyceride metabolism by regulating the expression of *GDF15*, *ADM2*, and *LDLR*.

## Results

### Paneth cells colocalize with TAS2R43 and TAS2R10^+^ cells, but goblet cells colocalize only with TAS2R43 in jejunal crypts of individuals with obesity.

**Figure 1:**
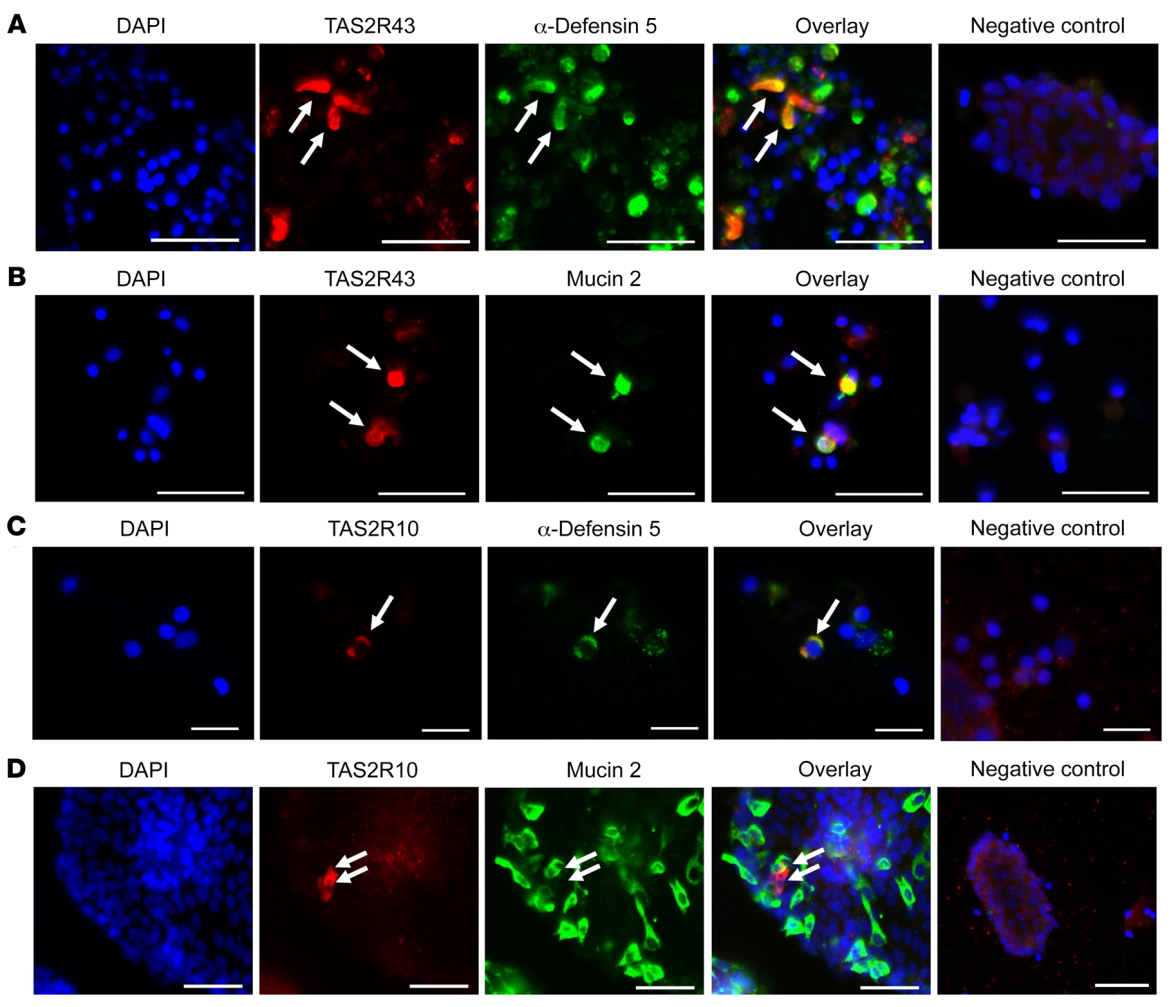
Colocalization of TAS2R43 and TAS2R10 with Paneth or goblet cells in primary crypts from patients with obesity. Representative double-immunofluorescence images of jejunal crypts from obese patients. Crypts were stained for TAS2R43 (**A **and** B**) or TAS2R10 (**C **and** D**) (red) and α-defensin 5 (in Paneth cells) or mucin 2 (in goblet cells) (green). Images in **A**–**C** show colocalization, whereas the images in **D** show a TAS2R10^+^ cell in close proximity to a goblet cell, but no colocalization. Nuclei were stained with DAPI (blue). Scale bars: 20 μm (**C**) and 50 μm (**A**, **B**, and **D**). Each colocalization study was repeated in crypts derived from at least 3 patients with obesity.

Double immunofluorescence staining of primary jejunal crypt cultures from patients with obesity showed that the bitter taste receptor TAS2R43 colocalized with α-defensin 5^+^ cells, a marker for Paneth cells (Figure 1A), and with mucin 2^+^ cells, a marker for goblet cells (Figure 1B). Of the 187 TAS2R43^+^ cells (*n =* 3 patients with obesity) analyzed, 42 cells stained positive for α-defensin 5 (22 %). In another set of 108 TAS2R43^+^ cells (*n =* 3 patients with obesity), 36 cells colocalized with mucin 2^+^ cells (33 %). We also confirmed the colocalization of TAS2R43 with Paneth cells and goblet cells in jejunal tissue sections derived from a lean individual (Supplemental Figure 1, A and B; supplemental material available online with this article; https://doi.org/10.1172/JCI144828DS1).

Colocalization studies of TAS2R10, a broadly tuned TAS2R, showed that 8 of 54 TAS2R10 immunoreactive cells, stained positive for α-defensin 5 (15 %, *n =* 8 patients with obesity; Figure 1C). We analyzed approximately 842 immunopositive mucin 2 cells from 3 different individuals with obesity, but no clear colocalization with TAS2R10 could be identified. However, we found several TAS2R10^+^ cells that were in close proximity to goblet cells (Figure 1D), indicating that goblet cells might be indirectly stimulated by TAS2R10 agonists.

### The bitter compounds DB and phenylthiocarbamide alter intracellular Ca^2+^ levels in primary jejunal crypts of obese individuals.

**Figure 2:**
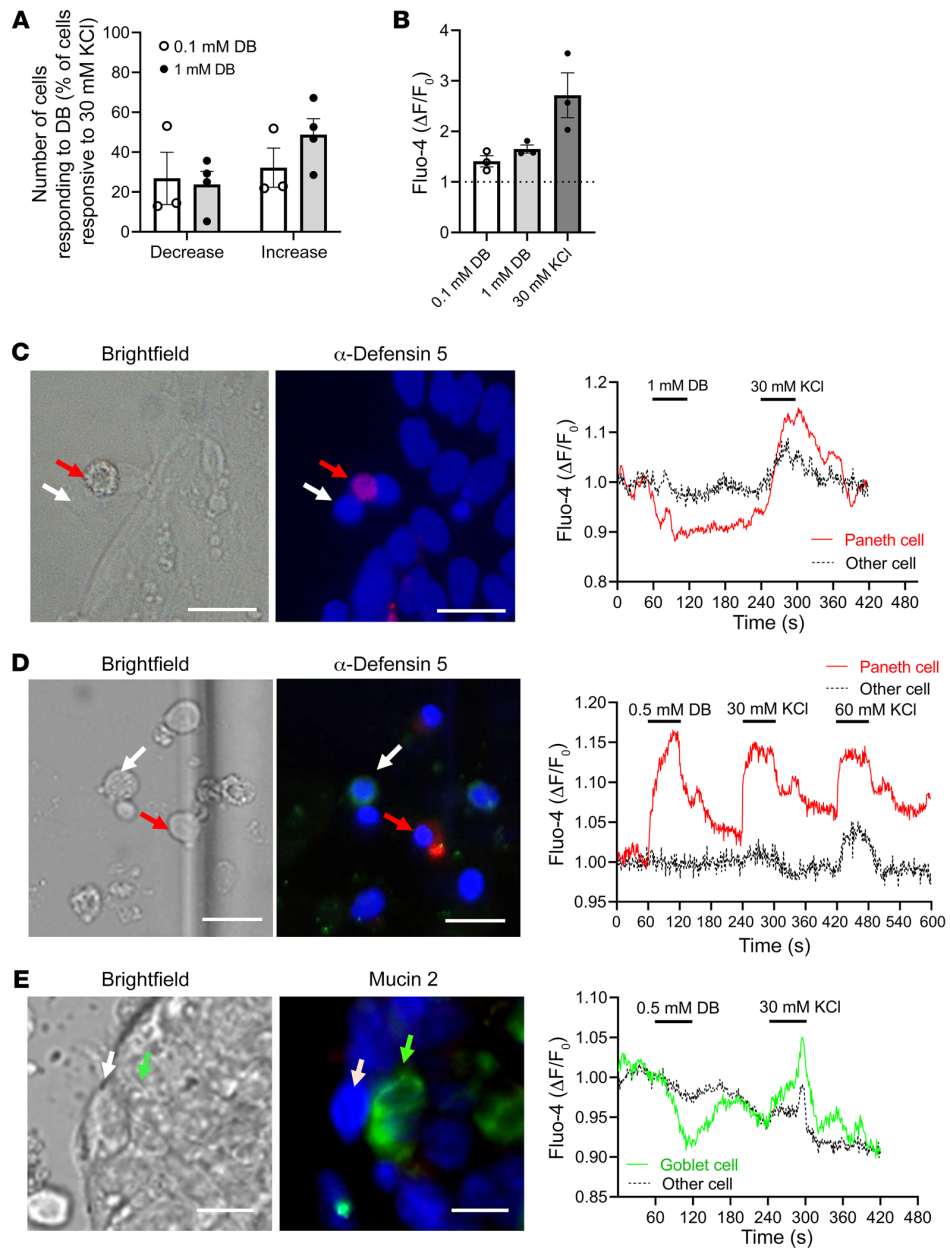
Intracellular Ca^2+^ changes in response to DB and immunostaining identification of cells in primary crypts from patients with obesity. (**A**) Percentage of 30 mM KCl-responsive primary jejunal cells from patients with obesity that responded to 0.1 mM or 1 mM DB administration with either a decrease or increase in intracellular Ca^2+^ levels. (**B**) Relative rise in fluorescence intensity in single cells from crypts of individuals with obesity treated with DB or KCl. Data represent the mean ± SEM. *n =* 3–4 subjects. (**C**–**E**) Images show the cells in bright-field, as well as immunofluorescence staining for α-defensin 5 (red) and mucin 2 (green). Nuclei were stained with DAPI (blue). Ca^2+^ changes in the regions of interest from the images in **C**–**E** are shown in the tracings. (**C**) Tracing of a Paneth cell responding to 1 mM DB with a Ca^2+^ rise and another unidentified, nonresponding cell. (**D**) Tracing of a Paneth cell responding to 0.5 mM DB with a Ca^2+^ increase and another unidentified, nonresponding cell. (**E**) Tracing of a goblet cell responding to 0.5 mM DB with a Ca^2+^ decrease and an unidentified, nonresponding cell. Scale bars: 20 μM.

DB, the most bitter chemical compound known, targets 8 different TAS2Rs (TAS2R4, -8, -10, -13, -30, -39, -43, -46), including TAS2R10 and TAS2R43. To determine whether bitter compounds can induce an immediate response in human jejunal crypt cells, we measured intracellular Ca^2+^ levels in response to DB exposure. After treatment with 0.1 mM DB, 27% ± 13% of all KCl-reactive (30 mM) cells responded with a Ca^2+^ decrease, whereas 32% ± 10% showed an increase (Figure 2A). With 1 mM DB, 24% ± 7% responded with a Ca^2+^ decrease and 49% ± 8% with an increase. The relative rise in fluorescence intensity amounted to 1.65 ± 0.08 (1 mM DB) compared with 2.72 ± 0.44 for 30 mM KCl (Figure 2B). To determine whether DB induced a Ca^2+^ change in Paneth or goblet cells, crypt cultures were occasionally immunostained after single-cell Ca^2+^ fluorescence imaging for α-defensin 5 or mucin 2. An α-defensin 5–immunoreactive cell responding to DB with either a decrease or an increase is shown in Figure 2, C and D, respectively. A mucin 2–immunoreactive cell responded with a Ca^2+^ decrease (Figure 2E). In addition, we also conducted the experiments with another bitter compound, 0.1 mM phenylthiocarbamide (PTC), which targets TAS2R38. The relative rise in fluorescence intensity was 1.63 ± 0.4 (0.1 mM PTC) compared with 1.69 ± 0.32 for 30 mM KCl (Supplemental Figure 2A). We identified a goblet cell that responded with an increase following PTC treatment (Supplemental Figure 2, B and C).

### DB induces an acute release of α-defensin 5 and lysozyme in crypts of individuals with obesity but not in lean individuals.

**Figure 3:**
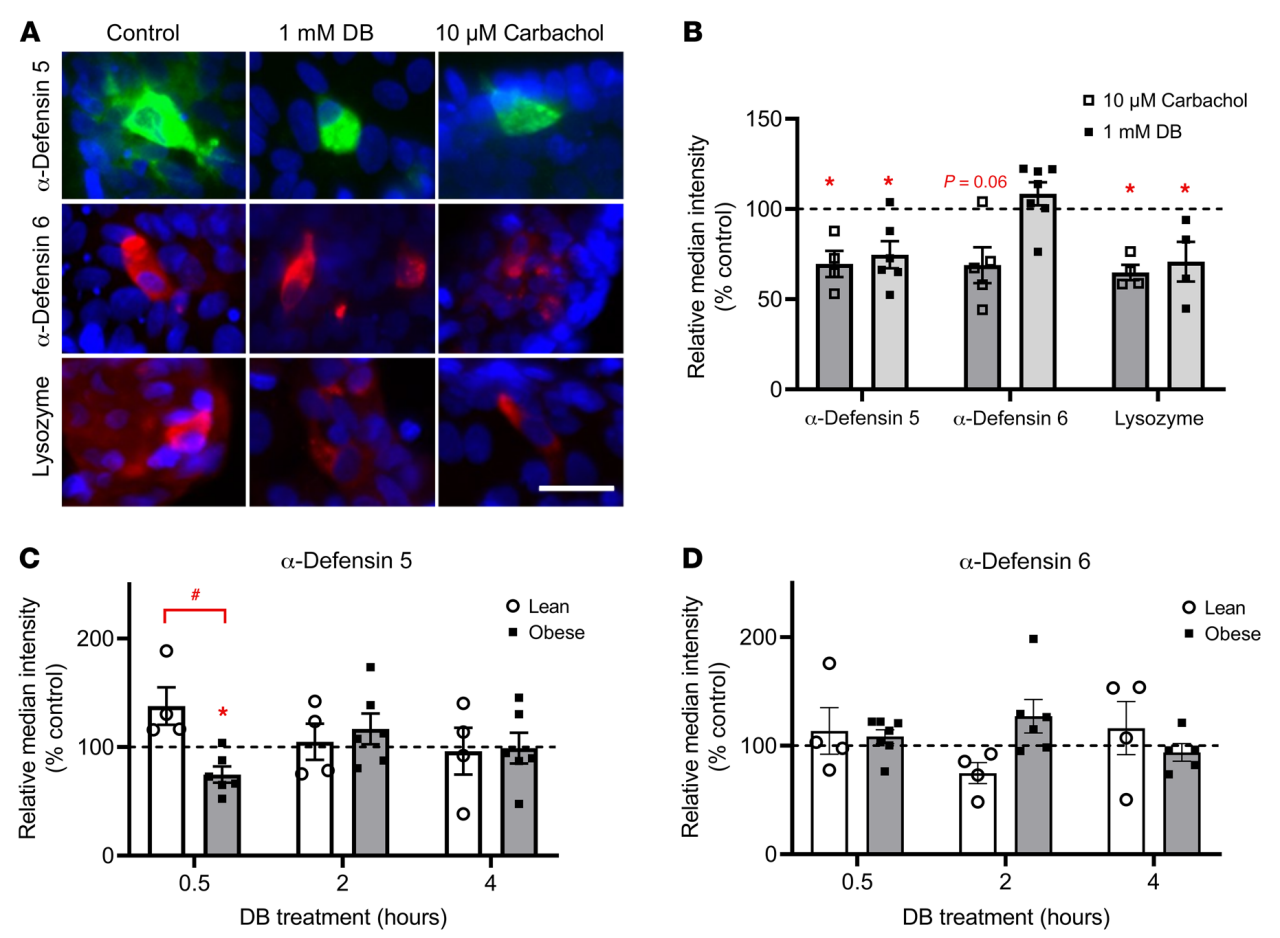
Quantitative analysis of the time-dependent effects of DB on protein expression in Paneth cells from lean and obese individuals. (**A**) Representative immunofluorescence staining for α-defensin 5, α-defensin 6, and lysozyme after a 30-minute treatment with DMEM, 1 mM DB, or 10 μM carbachol in jejunal crypts from a patient with obesity. Scale bar: 25 μm. (**B**) Quantification of immunostainings for α-defensin 5, α-defensin 6, and lysozyme after a 30-minute treatment of crypts from obese individuals with 1 mM DB or 10 μM carbachol, expressed relative to the control (DMEM) (*n =* 4–7). (**C **and** D**) Time-dependent effect (0.5–4 hours) of 1 mM DB treatment on the relative intensity of the immunostainings for α-defensin 5 and α-defensin 6 in jejunal crypts from lean and obese individuals (*n =* 4–7). Data represent the mean ± SEM, and single values are plotted. Statistical significance was determined using a mixed model with patient as the random effect. *P < 0.05, treatment versus control; ^#^*P <* 0.05, lean patients versus patients with obesity.

We evaluated the effect of DB on the release of antimicrobial peptides from Paneth cells by quantification of Paneth cell markers in immunostained images of crypts. Treatment of jejunal crypts from obese individuals with the positive control carbachol for 30 minutes reduced the median fluorescence intensity of α-defensin 5 by 34% ± 7% (*P <* 0.05), α-defensin 6 by 31% ± 10% (*P =* 0.06), and lysozyme by 36% ± 8% (*P <* 0.05), and hence induced the release of these proteins (Figure 3, A and B, and ref. 19). Treatment with 1 mM DB for 30 minutes also reduced α-defensin 5 expression by 37% ± 9% (*P <* 0.05) and lysozyme by 32% ± 9% (*P <* 0.05) but not α-defensin 6 expression in primary crypts from patients with obesity (Figure 3, A and B). No effects of DB on the release of these antimicrobial peptides in crypts from lean subjects were observed after 30 minutes or at later time points (Supplemental Figure 3 and Figure 3C). The effect of DB on the release of α-defensin 5 in jejunal crypts from individuals with obesity was an acute effect that was not observed after 2 or 4 hours of stimulation (Figure 3C). We observed no time-dependent effects of DB on α-defensin 6 protein expression in either study population (Figure 3D).

**Figure 4:**
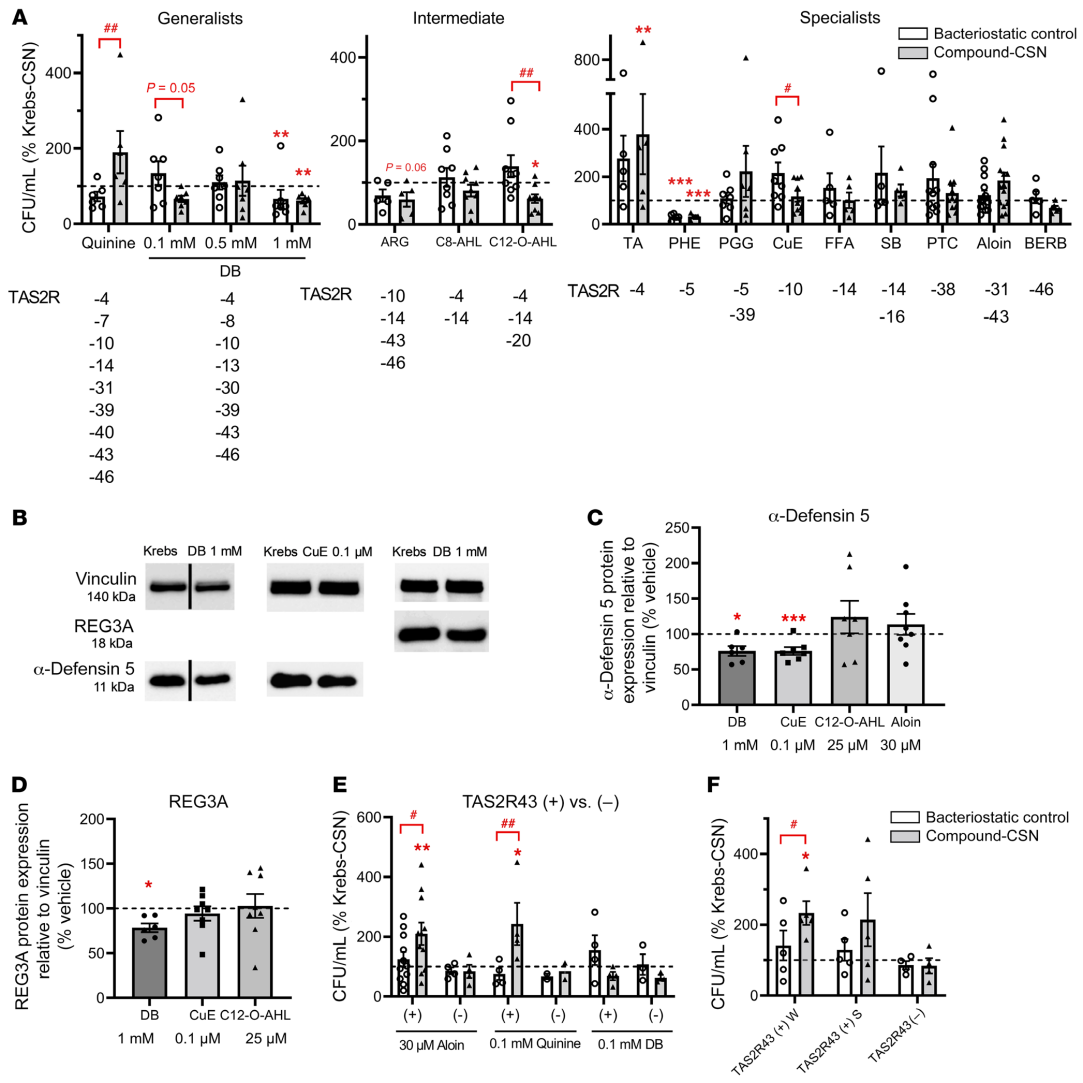
Role of TAS2R43 in the effect on *E*. *coli* growth of CSNs from patients with obesity *coli* growth of CSNs from patients with obesity stimulated with bitter compounds. (**A**) *E*. *coli* CFU following treatment with the supernatant of obese patients’ jejunal crypts (*n =* 5–14) stimulated for 30 minutes with bitter compounds (compound-CSN) or Krebs (Krebs-CSN). Crypt-independent effects of bitter agonists are shown as well (bacteriostatic control). All results are expressed as the percentage of Krebs-CSN with or without vehicle. Compounds were grouped by their TAS2R activation profile: generalists (>5 TAS2Rs); intermediates (3 or 4 TAS2Rs), and specialists (1 or 2 TAS2Rs). Test concentrations and abbreviations are given in Table 1. (**B**) Western blots showing expression of α-defensin 5 or REG3A in lysates from crypts from obese individuals. Crypts were treated for 30 minutes with bitter agonists or Krebs. Vertical line indicates that the lanes were run on the same gel but were noncontiguous. Summary of Western blot analyses for the effect on (**C**) α-defensin 5 or (**D**) REG3A protein expression (*n =* 6–8). (**E **and** F**) Effect of TAS2R43 deletion/amino acid polymorphisms. (**E**) Effect on *E*. *coli* growth of the supernatant of crypts from TAS2R43^+^ and TAS2R43^–^ patients with obesity. Crypts were stimulated for 30 minutes with the TAS2R43 agonists aloin (30 μM, *n =* 11 TAS2R43^+^, *n =* 4 TAS2R43^–^); quinine (0.1 mM, *n =* 4 TAS2R43^+^, *n =* 2 TAS2R43^–^); or DB (0.1 mM, *n =* 4 TAS2R43^+^, *n =* 3 TAS2R43^–^). (**F**) Effect on *E*. *coli* growth of the supernatant of crypts from obese patients with a TAS2R43 genotype that is highly sensitive (*n =* 5 TAS2R43^+^ W); mildly sensitive (*n =* 5 TAS2R43^+^ S); or not sensitive (*n =* 4 TAS2R43^–^) to aloin. Data represent the mean ± SEM, and single values are plotted. Statistical significance was determined using a mixed model with patient as the random effect and, for Western blot analysis, a paired Student’s *t* test with Bonferroni-Holm adjustment. *P < 0.05, **P < 0.01, and ***P < 0.001, versus Krebs-CSN or the vehicle control (100% control); ^#^*P <* 0.05 and ^##^*P <* 0.01, versus the bacteriostatic control.

Western blot analysis of cell lysates from primary crypts from individuals with obesity treated for 30 minutes with 1 mM DB showed a reduction in α-defensin 5 protein expression of 23.9% ± 6.8% compared with vehicle treatment and confirmed the immunostaining results (Figure 4, B and C).

### Bitter agonists selectively induce the release of molecules from jejunal crypts of individuals with obesity that either inhibit or stimulate E. coli growth.

We studied the effect of the supernatant from jejunal crypts of patients with obesity stimulated for 30 minutes with bitter agonists on *E*. *coli* growth by counting the number of CFU. Because of the limited effects of DB on defensin expression in crypts from lean patients, this screening experiment was performed only with cultures from patients with obesity.

We compared the time-dependent effects of the supernatant from obese individuals’ jejunal crypts stimulated with 1 mM DB (DB–crypt supernatant [DB-CSN]) versus Krebs buffer (control, Krebs-CSN) on the growth of *E*. *coli*. To exclude the possibility that the test compounds themselves had crypt-independent effects on *E*. *coli* growth, test compounds were added to Krebs-CSN after removal from the crypts (bacteriostatic control, Krebs-CSN plus DB). *E*. *coli* were growing continuously over a 24-hour period in the supernatant from crypts stimulated with Krebs buffer (Krebs-CSN; Supplemental Figure 4). In comparison with Krebs-CSN, DB-CSN inhibited the growth of bacteria in a time-dependent manner (*P <* 0.01; Supplemental Figure 4). However, we observed a similar inhibition (*P <* 0.05) with Krebs-CSN plus DB, indicating direct bacteriostatic effects induced by the bitter compound itself. The number of CFU/mL was reduced by 51% ± 9% for DB-CSN and by 75% ± 10% for Krebs-CSN plus DB after 24 hours, but these effects were not significantly different from each other.

In order to find bitter compounds without bacteriostatic effects, we tested several other synthetic and natural bitter compounds as well as bitter bacterial quorum–sensing molecules that activate TAS2Rs that are well expressed in the gut. The relative mRNA expression of several TAS2Rs in jejunal crypts from individuals with obesity is shown in Supplemental Figure 5. Among these, we found that TAS2R14 was the most highly expressed and that TAS2R4, -5, -10, -13, -20, -30, -43 were expressed at middle-range levels, while TAS2R7, -8, -31, -38, -39, -40, -46 expression levels were rather low. Expression levels in primary crypts did not significantly differ between lean and obese individuals. Bitter compounds were divided on the basis of their TAS2R activation profile into generalists (activating >5 TAS2Rs), intermediates (activating 3–4 TAS2Rs), or specialists (activating 1 or 2 TAS2Rs) (see overview in Table 1 and refs. 20–23). Although 1 mM DB was bacteriostatic for *E*. *coli*, 0.1 mM was not and induced a reduction (*P =* 0.05) in *E*. *coli* CFU/mL compared with its bacteriostatic control (Figure 4A). Another generalist showed the opposite effect: quinine-CSN stimulated (*P <* 0.01) *E*. *coli* growth compared with its bacteriostatic control.

Of the intermediates, only the supernatant of crypts stimulated with the quorum-sensing molecule *N*-(3-oxododecanoyl)-l-homoserine lactone C12-O-AHL (25 μM, activating TAS2R4, -14, and -20) induced a significant (*P <* 0.05) decrease compared with Krebs-CSN and its bacteriostatic control (*P <* 0.01; Figure 4A). *N*-octanoyl-l-homoserine lactone (C8-AHL, 100 μM) activating TAS2R4 and TAS2R14, but not TAS2R20 (21), showed no effects at all. This indirectly indicates an important role for TAS2R20 in the antibacterial effects of C12-O-AHL. Arglabin (ARG, 10 μM) activates 3 TAS2Rs in common with DB. Arglabin-CSN showed a trend (*P =* 0.06) toward reducing *E*. *coli* growth compared with Krebs-CSN, but it was not significantly different from the reduction induced by the bacteriostatic control.

We tested several bitter specialists, which gave us a clearer indication of the TAS2Rs involved in the release of molecules from primary jejunal crypts that alter bacterial growth (Figure 4A). Treatment of crypts with the bile acid taurocholic acid (TA, 0.5 mM), which activates TAS2R4, induced the release of molecules that increased (*P <* 0.01) *E. coli* growth compared with Krebs-CSN, but not in comparison with its bacteriostatic control. 1,10-Phenanthroline (PHE, 0.1 mM), a TAS2R5 agonist, is bacteriostatic for *E*. *coli* (*P <* 0.001), therefore, no additional effects could be identified. The TAS2R10 agonist cucurbitacin E (CuE, 0.1 μM) decreased (*P <* 0.05) bacterial growth in comparison with its bacteriostatic control but had no effect in comparison with Krebs-CSN. All other bitter compounds such as PTC (1 mM, activating TAS2R38); penta-*O*-galloyl-β-d-glucose (PGG, 10 μM, activating TAS2R5 and TAS2R39); flufenamic acid (FFA, 30 μM, activating TAS2R14); sodium benzoate (SB, 1 mM, activating TAS2R14 and TAS2R16); and berberine (BERB, 10 μM, activating TAS2R46) had no direct or indirect effect on *E*. *coli* growth (Figure 4A).

### Western blot analysis confirms the release of antimicrobial peptides by DB and CuE after treatment of crypts from patients with obesity.

The bitter compounds that inhibited *E*. *coli* growth had differential effects on the release of antimicrobial peptides. Western blot analysis showed that DB (1 mM) and CuE (0.1 M), but not C12-O-AHL (25 M), significantly reduced the expression of α-defensin 5 in the crypt lysates from patients with obesity (Figure 4, B and C). Aloin had no effect on the protein expression of α-defensin 5. The protein expression of another microbial peptide, regenerating islet–derived protein 3 α (REG3A), was only decreased with DB (1 mM) but not with CuE or C12-O-AHL (Figure 4, B and D).

### TAS2R43 determines the effect of aloin and quinine on bacterial growth.

Research in TAS2Rs has been hampered by a lack of specific TAS2R antagonists. We took advantage of a whole gene deletion polymorphism for TAS2R43 that exists in approximately 33% of the population (17) to compare the effects of TAS2R43 agonists in obese individuals with (TAS2R43^+^) and without (TAS2R43^–^) TAS2R43.

We tested the effect of aloin in crypts from 14 patients with obesity, of whom 21% had a deletion polymorphism. Aloin-CSN (30 μM) showed no significant effect on *E*. *coli* growth when all samples (TAS2R43^+^ and TAS2R43^–^) were analyzed together. However, when data were split up according to the patients’ genotype, aloin increased *E*. *coli* growth in comparison with Krebs-CSN (*P <* 0.01) and its bacteriostatic control (*P <* 0.05) in TAS2R43^+^ patients but not in TAS2R43^–^ patients (Figure 4E). TAS2R43 patients are further characterized by an amino acid polymorphism that determines their oral taste sensitivity to aloin (18). Individuals with a tryptophan in position 35 of TAS2R43 (TAS2R43^+^ W) are very sensitive to the bitterness of aloin, whereas those who have a serine at position 35 (TAS2R43^+^ S) are less sensitive to aloin (18). In samples from TAS2R43^+^ W patients with obesity (43%, *n =* 6), aloin-CSN significantly (*P <* 0.05) increased *E*. *coli* growth compared with Krebs-CSN and its bacteriostatic control (Figure 4F). In contrast, in samples from less sensitive TAS2R43^+^ S patients (36%, *n =* 5–6), aloin-CSN showed only a weak trend (*P <* 0.1) for an increase, and in the TAS2R43^–^ group (21%, *n =* 3–4), we observed no effect at all. These findings indicate that, similar to the oral cavity also in the gut, the sensitivity of TAS2R43 toward aloin was altered by its genotype.

Since DB and quinine also target TAS2R43, in addition to several other TAS2R subtypes, we also compared their effects in patients with or without TAS2R43. We found that TAS2R43 was not involved in the effect of DB, but it was involved in the growth-enhancing effect induced by quinine (Figure 4E). Therefore, activation of TAS2R43 induced the release of molecules that enhanced *E*. *coli* growth.

### Aloin induces the release of the mucus glycoprotein CLCA1 in TAS2R43^+^ patients.

**Figure 5:**
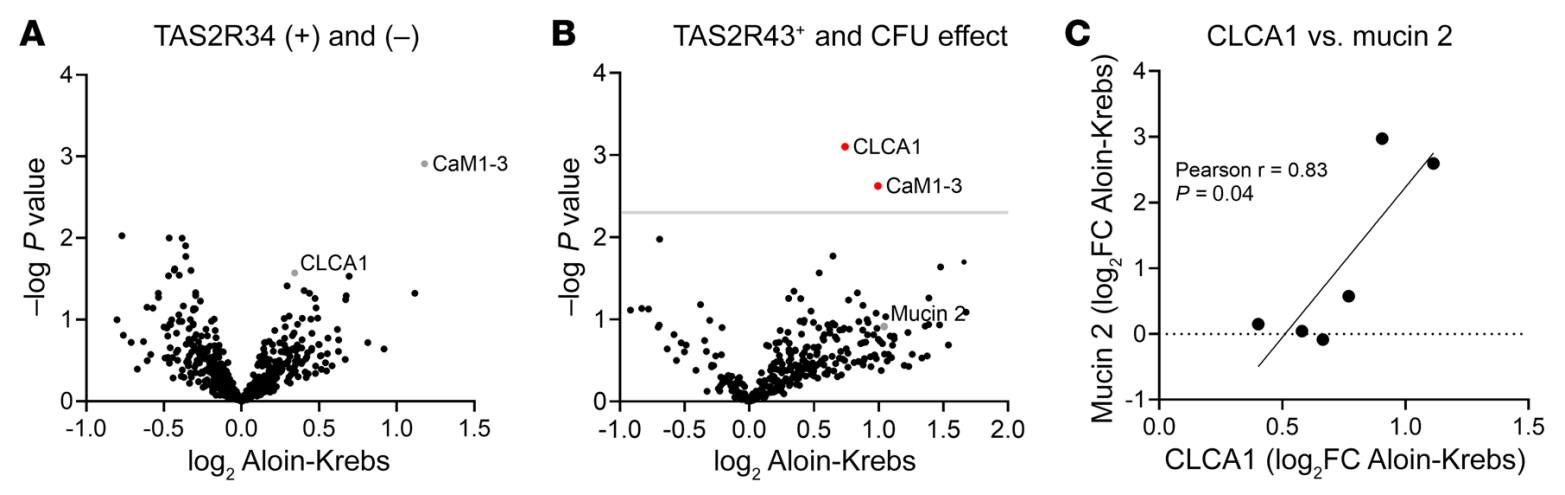
Proteomics analysis of the supernatant of jejunal crypts from patients with obesity stimulated with aloin, according to TAS2R43 genotype and effect on *E*. *coli* growth measured by CFU. (**A**) Volcano plot showing up- and downregulated proteins (log_2_ aloin-Krebs) in the data set of all supernatant samples (*n =* 14) from crypts stimulated with 30 μM aloin (aloin-CSN), including all TAS2R43 genotypes. No significant difference in secreted proteins was observed with a permutation-based FDR of 0.05, S0 = 0, using a paired, 2-sample Student’s *t* test. (**B**) Volcano plot showing 2 significantly upregulated proteins (CLCA1, CALM1–3) in a data set (*n =* 6) including only the aloin-CSN samples from TAS2R43^+^ patients with obesity that stimulated *E*. *coli* growth in the CFU assay. The same statistical calculations were used as in **A**. (**C**) Positive correlation of the log_2_ FC of the mucus proteins CLCA1 and mucin 2.

To identify how aloin-CSN increased *E*. *coli* growth in the CFU assay, we analyzed proteins in the CSN samples by mass spectrometry. A comparison of the log_2_ label-free quantification (LFQ) of aloin-CSN versus the log_2_ LFQ of Krebs-CSN for all samples including all TAS2R43 genotypes (*n =* 14 patients, filtering for at least 85% data completeness, 584 proteins) using a paired Student’s *t* test identified 19 proteins with a *P* value of less than 0.05 and log_2_ ± 0.05. However, none was significant after adjustment of the *P* value by a permutation-based FDR (0.05, minimal fold change [S0] = 0; Figure 5A). However, analysis of only the samples that stimulated bacterial growth in the CFU assay and that were from TAS2R43^+^ patients (*n =* 6) revealed that 2 proteins, the calcium-activated chloride channel regulator 1 (CLCA1) (log_2_ Aloin-Krebs 0.74, *q* = 0) and calmodulins 1–3 (CaM1–3) (log_2_ Aloin-Krebs 0.99, *q* = 0.036), were significantly increased after aloin treatment (Figure 5B). Calmodulin is a Ca^2+^ sensor protein that regulates many Ca^2+^-dependent enzymes (24). CLCA1 is a glycoprotein produced by goblet cells and a major component of the intestinal mucus layer (25). It also functions as a protease that can cleave the N-terminal part of the mucus structural component mucin 2 (26). Mucin 2 was not significantly increased by aloin treatment. However, the log_2_ LFQ of aloin minus the log_2_ LFQ of Krebs (log_2_ aloin-Krebs CSN) for mucin 2 and CLCA1 correlated with each other (Figure 5C).

### DB alters mRNA expression of α-defensins in jejunal crypts from lean and obese individuals independent of TAS2R43.

All previous experiments were performed after acute exposure (30 min) of primary crypts to bitter compounds to avoid breakdown of the released defensins and mucins. We next evaluated the effect of prolonged (4 h) incubation of crypts with bitter compounds on the mRNA expression of innate immune markers.

**Figure 6:**
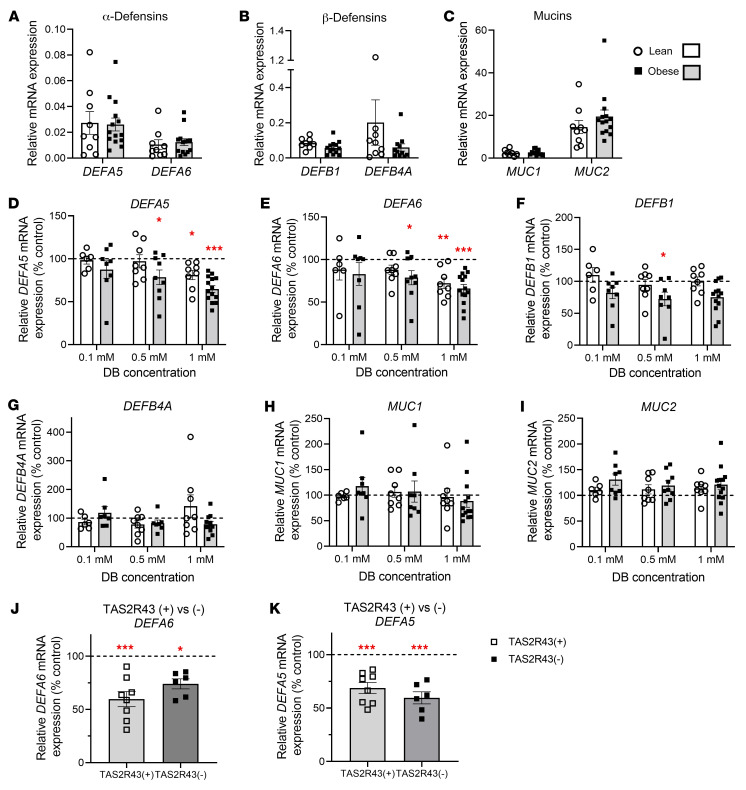
Changes in the mRNA expression of defensins and mucins in response to DB in crypts from lean and obese individuals. (**A**–**C**) Comparison of the basal mRNA expression of (**A**) α-defensins (*DEFA5* and *DEFA6*), (**B**) β-defensins (*DEFB1* and *DEFB4A*), and (**C**) mucins (*MUC1*, *MUC2*) in nonstimulated jejunal crypts from lean individuals (*n =* 8–9) and patients with obesity (*n =* 11–14). (**D**–**I**) Concentration-dependent effects of a 4-hour stimulation of jejunal crypts from lean (*n =* 6–8) and obese (*n =* 7–14) subjects with DB (0.1–1 mM) on the mRNA expression of (**D **and** E**) α-defensins, (**F **and** G**) β-defensins, and (**H** and **I**) mucins. (**J **and** K**) Role of TAS2R43 in the effect of 1 mM DB on (**J**) *DEFA6* and (**K**) *DEFA5* mRNA expression in jejunal crypts from TAS2R43^+^ (*n =* 8) and TAS2R43^–^ (*n =* 6) patients with obesity. *RPS18* and *GAPDH* were used as endogenous controls. Data represent the mean ± SEM, and single values are plotted. Statistical significance was determined using a mixed model with patient as the random effect. *P < 0.05, **P < 0.01, and ***P < 0.001 versus control (DMEM).

We found that basal mRNA expression of α-defensins (*DEFA5*, *DEFA6*), β-defensins (*DEFB1*, *DEFB4A*) and mucins (*MUC1*, *MUC2*) in the jejunum did not differ between lean and obese individuals (Figure 6, A–C). DB decreased the mRNA expression of *DEFA5* in a concentration-dependent manner in jejunal crypts from individuals with obesity (concentration effect: *P <* 0.001) and showed a trend toward decreased expression in lean individuals (concentration effect: *P =* 0.06) (Figure 6D). We observed similar effects of DB on *DEFA6* mRNA expression (concentration effect in lean individuals: *P <* 0.05; individuals with obesity: *P <* 0.01; Figure 6E). However, DB decreased (concentration effect: *P <* 0.05; Figure 6F) *DEFB1* mRNA expression and tended to decrease (concentration effect: *P =* 0.05; Figure 6G) *DEFB4A* mRNA expression in patients with obesity but not in lean individuals. DB treatment did not affect the expression of *MUC1* or *MUC2* (Figure 6, H and I).

In total, 38% of patients with obesity were TAS2R43^–^. The TAS2R43 deletion polymorphism did not affect the basal expression of the innate immune markers in jejunal crypts (data not shown). The broadly tuned TAS2R agonist DB decreased *DEFA6* mRNA expression in crypts of TAS2R43^+^ individuals by 43.4% ± 5.9 % (*P <* 0.001) and to a lesser extent in TAS2R43^–^ individuals with obesity (26.7% ± 8.8%, *P <* 0.05; Figure 6J). The magnitude of the decrease (*P <* 0.001) in *DEFA5* mRNA expression induced by DB did not differ between crypts from TAS2R43^+^ and TAS2R43^–^ patients with obesity (Figure 6K). The TAS2R43 deletion polymorphism also did not have an impact on the effect of DB on *DEFB1*, *DEFB4A*, *MUC1*, or *MUC2* mRNA expression (data not shown).

### DB treatment alters the mRNA expression of immune factors and metabolic regulators.

We performed transcriptomic analysis by RNA-Seq in order to analyze how 4 hours of treatment with DB or aloin affected the transcriptome of human jejunal crypts compared with DMEM-treated crypts.

**Figure 7:**
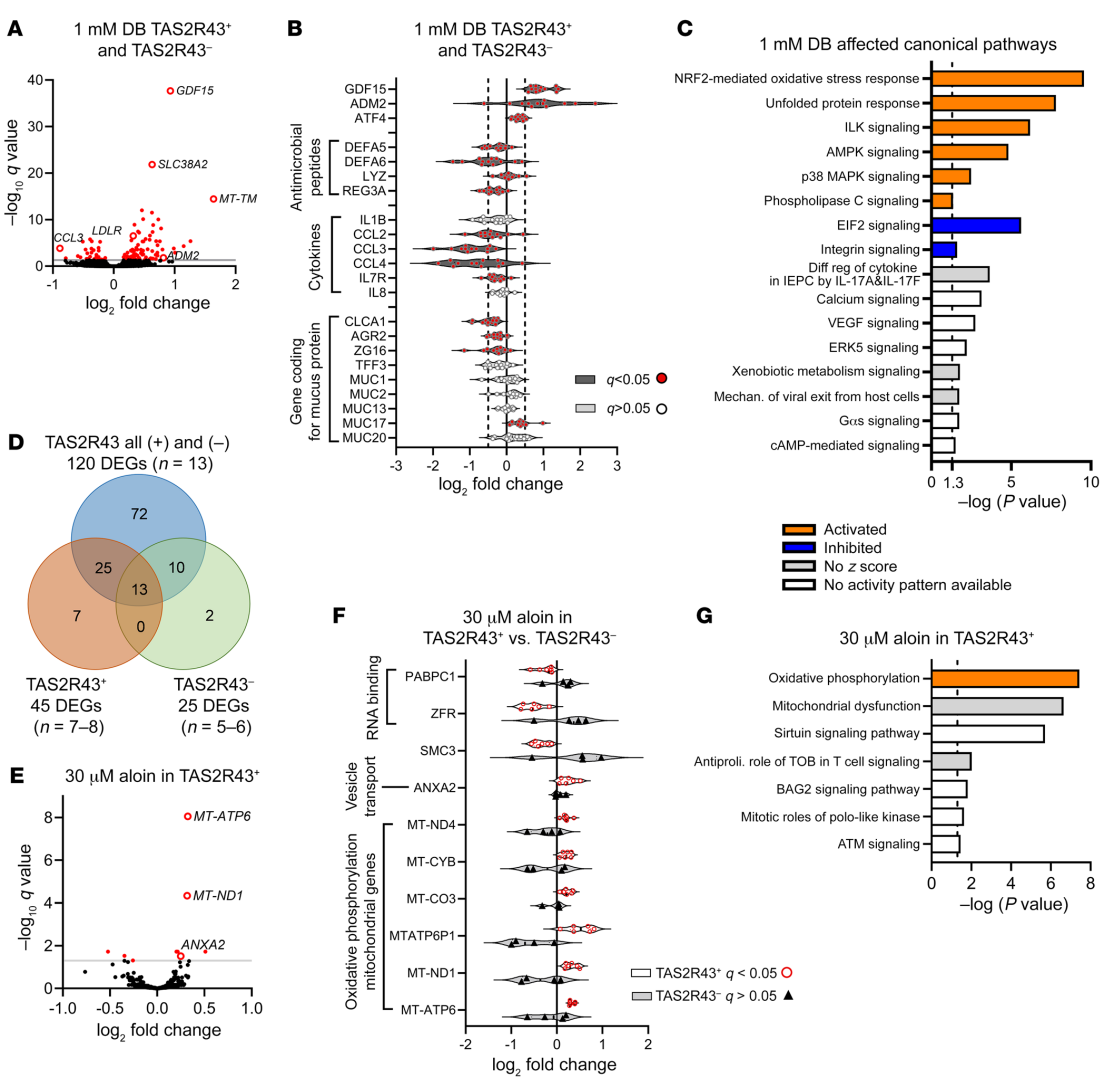
Transcriptomics analysis of jejunal crypts from patients with obesity stimulated with DB or aloin. Significantly DEGs revealed by transcriptomics analysis of human primary jejunal crypts from patients with obesity (*n =* 5–13) after 4 hours of treatment with the bitter compounds DB or aloin. (**A**) Volcano plot showing the log_2_ FCs of the detected genes after treatment of crypts with 1 mM DB versus DMEM (*n =* 13). Significantly DEGs are indicated in red. (**B**) Selected DEGs of Paneth cell markers (antimicrobial peptides), cytokines, and goblet cell markers (mucus proteins) after treatment with 1 mM DB. (**C**) Canonical pathways affected by 1 mM DB treatment, identified by analyzing the RNA-Seq data with IPA. Diff reg, differential regulation; Mechan., mechanism. (**D**) Venn Diagram comparing the amount of significantly DEGs identified in the data sets that included either all TAS2R43^+^ and TAS2R43^–^ or only TAS2R43^+^ or only TAS2R43^–^ genotypes. (**E**) Volcano plot showing the log_2_ FCs of the detected genes after treatment of crypts with 30 μM aloin or DMEM (*n =* 6–8). Significantly DEGs are indicated in red. (**F**) DEGs after treatment with 30 μM aloin in crypts from TAS2R43^+^ (*n =* 6) compared with TAS2R43^–^ (*n =* 4) patients with obesity. (**G**) IPA identification of the canonical pathways that were affected in jejunal crypts after treatment with 30 μM aloin. Antiproli., antiproliferative.

Treatment of crypts from patients with obesity, independent of TAS2R43 genotype, with 1 mM DB resulted in 120 DEGs compared with control-treated (DMEM-treated) crypts, as identified by an adjusted *P* value (*q*) of less than 0.05 (Figure 7A and Supplemental Figure 6). The most significantly affected and upregulated genes were growth differentiation factor 15 (*GDF15*) (log_2_ fold change [FC] of 0.93, *q* < 0.001); the amino acid transporter *SLC38A2* (log_2_ FC of 0.63, *q* < 0.001); and the mitochondrially encoded transfer RNA methionine (*MT-TM*) (log_2_ FC of 1.64, *q* < 0.001) (Figure 7A). In addition, downregulation of *DEFA5* (log_2_ FC of –0.25, *q* = 0.01) and *DEFA6* (log_2_ FC of –0.45, *q* = 0.03) confirmed the result from the previous quantitative PCR (qPCR) study (Figure 7B). Another antimicrobial peptide, *REG3A* (log_2_ FC of –0.33, *q* < 0.001), was also decreased by DB treatment, as were the mucus protein–encoding genes (27) *CLCA1* (log_2_ FC of –0.51, *q* < 0.001); *AGR2* (log_2_ FC of –0.25, *q* < 0.001); and *ZG16* (log_2_ FC of –0.27, *q* < 0.05). Of the mucins, we found that only the expression of *MUC17* (log_2_ FC of 0.39, *q* < 0.001) was increased. We observed the most pronounced decrease for the chemokines *CCL3* (log_2_ FC of –0.88, *q* < 0.001) and *CCL4* (log_2_ FC of –0.79, *q* < 0.05). Ingenuity Pathway Analysis (IPA) revealed that these 2 molecules, together with the downregulation of *CCL2* (log_2_ FC of –0.43, *q* < 0.01), are associated with differential regulation of cytokine production in intestinal epithelial cells by IL-17A and IL-17F (ratio: 0.13, *z* score: could not be calculated, –log *P* value = 3.65; Figure 7C).

In addition, 1 mM DB treatment activated the NRF2-mediated oxidative stress response, the unfolded protein response, and ILK, AMPK, p38 MAPK, and phospholipase C signaling, while it inhibited integrin and EIF2 signaling pathways (Figure 7C). The transcription factor 4 (*ATF4*) was significantly upregulated (log_2_ FC of 0.34, *q* < 0.001) and was identified as an activated upstream regulator in the IPA analysis, with the highest *z* score of 3.486 (bias-corrected *z* score: 1.93, *P <* 0.001). ATF4 is a known regulator of the uncoupled protein response and mediates *GDF15* expression (28). We confirmed the RNA-Seq data by qPCR and showed that DB, but not aloin, increased (*P <* 0.001) GDF15 expression in a concentration-dependent manner in jejunal crypts from patients with obesity (Supplemental Figure 7).

A global correlation matrix of the 120 DEGs identified after 1 mM DB treatment in individuals with obesity revealed that *GDF15* strongly correlated with another mitokine, adrenomedullin 2 (*ADM2*) and the LDL receptor (*LDLR*) (Supplemental Figure 8).

Analysis of mRNA expression in crypts derived from lean patients (TAS2R43^+^, *n =* 4) revealed that only 8 DEGs could be identified after treatment with 1 mM DB, which overlapped with the DEGs identified in the obese data set. *GDF15* (log_2_ FC of 1.09, *q* < 0.001) as well as several mitochondrial genes (*MT-TL1*, *MT-TF*, *MT-TM*, *MT-TP*, *MT-TS2*, *MT-ND6*) were upregulated, whereas *CCL2* (log_2_ FC of –1.22, *q* < 0.05) was downregulated (Supplemental Figure 9A).

Taken together, these findings indicate that, in jejunal crypts from patients with obesity, DB altered the expression of immune factors (antimicrobial peptides, mucins, cytokines) and induced a mild stress response that led to the upregulation of mitokine genes that control food intake (*GDF15*, *ADM2*) (28, 29) and lipoprotein homeostasis (*LDLR*).

### TAS2R43 genotype influences the identification of DEGs after DB treatment in RNA-Seq data.

A separate analysis of mRNA expression in TAS2R43^+^ (*n =* 7–8) and TAS2R43^–^ (*n =* 5–6) patients after 1 mM DB treatment resulted in 45 and 25 significantly (*q* < 0.05) DEGs, respectively. By comparing the significantly up- and downregulated DEGs in the data set “all” (TAS2R43^+^ and TAS2R43^–^) with those resulting from the TAS2R43^+^ and TAS2R43^–^ data sets, we identified an overlap of 13 DEGs between the 3 groups (Figure 7D). In all 3 groups, we found that *GDF15* was the most significantly expressed DEG, indicating that the increase in expression of that gene is independent of the TAS2R43 genotype. A total of 7 DEGs (*RNU4ATAC*, *PABPC1*, *MT-TV*, *GBP2*, *CXCL5*, *PTPRC*, and *NUCKS1*) were only identified in the TAS2R43^+^ group (Supplemental Figure 9B). Therefore, TAS2R43 might be involved in the regulation of RNA metabolism (*RNU4ATAC*, *PABPC1*, *MT-TV*) and in chemokine or cytokine signaling (*GBP2*, *CXCL5*, *PTPRC)*.

### TAS2R43 regulates the effect of aloin on oxidative phosphorylation.

Treatment (4 h) of jejunal crypts from TAS2R43^+^ patients with obesity with the TAS2R43 agonist aloin (30 M, *n =* 6) versus vehicle (*n =* 8) resulted in the identification of 10 DEGs (Figure 7, E and F). No DEGs were identified in TAS2R43^–^ patients (control, *n =* 5; aloin-treated, *n* = 4). These findings indicate an involvement of TAS2R43 in the regulation of these genes. The volcano plot shows that the most significantly increased gene was *MT-ATP6* (log_2_ FC of 0.32, *q* < 0.001). Together with 3 other significantly upregulated mitochondrial genes, *MT-ATP6* belongs to the oxidative phosphorylation pathway (ratio: 0.0377, *z* score: 2, –log *P* value: 7.44). *ANXA2* is part of the BAG2 signaling pathway (ratio: 0.0238, *z* score: not calculated, –log *P* value: 1.82), but is also known to be involved in secretory vesicle exocytosis (ref. 30 and Figure 7G). Two downregulated genes, *ZFR* and *PABPC1*, are involved in RNA binding and are therefore involved in the translation of proteins (Figure 7F and ref. 31).

Our analysis of crypts from TAS2R43^+^ lean individuals treated with aloin (control, *n =* 4; aloin-treated, *n =* 6) did not reveal any DEGs.

## Discussion

Innate immune responses initiated by TAS2R activation were first described in the respiratory tract (13, 32, 33) and recently also in mouse gingiva (34). Although activation of TAS2Rs in the small intestine has been previously associated with the release of gut hormones (4), the finding in mice that Paneth cells and goblet cells express a bitter taste receptor (11, 12) indicates that bitter compounds are also involved in the first line of defense in the gut. Here, we report for the first time to our knowledge that bitter compounds can induce the acute release of antimicrobial peptides from Paneth cells and mucus proteins from goblet cells in the human gut epithelium to alter bacterial growth as well as mitokine expression that alters metabolism.

We detected the expression of TAS2R43 and TAS2R10, which are targeted by the bitter compound DB, on Paneth cells and/or goblet cells. Bitter agonists affected intracellular Ca^2+^ levels in human intestinal crypt cells, including Paneth and goblet cells, indicating that TAS2Rs have a functional role in these cell types.

In jejunal crypts from patients with obesity, but not in those from lean individuals, DB induced an acute decrease in α-defensin 5 protein expression. It is currently unclear why jejunal crypts from patients with obesity are more sensitive to bitter-induced Paneth cell degranulation than are lean controls, since our immunofluorescence studies confirmed the expression of TAS2Rs on Paneth and goblet cells. We found no difference in mRNA expression levels of several TAS2Rs, including those targeted by DB, in jejunal crypts from lean or obese individuals. However, we cannot exclude a possible alteration of TAS2Rs expression at the level of the Paneth cells, since experiments were performed with a mixed epithelial cell preparation.

Further, it is important to point out that we did not test the effect of DB on the release of other antimicrobial peptides or of other bitter compounds in lean primary cultures, nor did we perform any further functional studies. We therefore cannot claim that this acute defense mechanism is not functional in lean subjects but only that these individuals are less sensitive to bitter compounds. This is also revealed by the transcriptional changes in the expression of innate immune and mitokine markers after prolonged stimulation with DB, which were similar in both patient populations but less pronounced in lean patients. The unfolded protein response pathway (EIF2 signaling), which regulates proinflammatory cytokine expression and bacterial invasion, was markedly upregulated in patients with obesity compared with that observed in lean patients (data not shown). Activation of this pathway has previously been shown to affect Paneth cell function in patients with obesity (15). It is likely that TAS2R-induced antimicrobial peptide release may synergize with this downstream signaling pathway in patients with obesity, but not in lean patients, to further enhance Paneth cell degranulation. Further studies are required to identify possible crosstalk. Obesity is characterized by a “leaky gut” due to perturbed gut integrity and permeability (35). This “leaky gut” favors bacterial translocation and leakage of bacteria, metabolites, microbial toxins, and luminal and food particles into the circulation. From a physiological point of view, we can only speculate that the increased sensitivity that induces the release of antimicrobial peptides in response to bitter-tasting toxins in patients with obesity may serve as a feedback mechanism to compartmentalize microbial antigens and toxins that inhabit the intestine. This may prevent the translocation of these antigens and toxins and counteract further susceptibility of the host to systemic inflammation.

Since DB induced an acute release of antimicrobial peptides in crypts from obese individuals, we investigated whether this was of physiological relevance and could alter *E*. *coli* growth. Indeed, the supernatant of crypts treated with 0.1 mM DB reduced the growth of *E*. *coli*, but a higher dose (1 mM) of DB had crypt-independent effects and reduced *E*. *coli* growth by itself. Similar effects were observed with the synthetic bitter compound 1,10-phenanthroline (TAS2R5). To our knowledge, no bacteriostatic or bactericidal effects of DB against *E*. *coli* have been reported, but the effects of antimicrobial peptides in an experimental setting may highly depend on the type of bacterium chosen. For instance, no bacteriostatic effects were reported with high concentrations (10 mM) of DB against *P. aeruginosa* (13). In general, the positive charge of a molecule may facilitate its binding to the negatively charged bacterial cell surface in order to affect bacterial activity (36).

On the basis of TAS2R profiling with more specific bitter agonists, we conclude that TAS2R10, which is well expressed in the gut, is one of the most plausible candidates via which DB elicits its antibacterial effects. Our studies showed a direct stimulation of Paneth cells by DB, based on the expression of TAS2R10 and the DB-induced Ca^2+^ changes in Paneth cells. Further, the inhibitory effect of DB on bacterial growth was mimicked with the TAS2R10 agonist CuE. Western blot studies showed that DB induced the release of α-defensin 5 and REG3A, whereas CuE only affected α-defensin 5 release. The generalist DB is activating 7 other TAS2Rs beyond TAS2R10, including TAS2R43, which was expressed on Paneth cells. Nevertheless, the TAS2R43 agonist aloin did not affect α-defensin 5 release. However, the possibility of effects at higher concentrations of aloin or during prolonged stimulation cannot be excluded.

Possible candidates for the effect of DB on REG3A release are TAS2R4, TAS2R13, TAS2R30, and TAS2R43, which were highly expressed in the primary crypts. The finding that C12-O-AHL (TAS2R4, -14, -20), but not C8-AHL (TAS2R4, -14), inhibited *E*. *coli* growth suggested an important role for TAS2R20, but C12-O-AHL treatment did not alter α-defensin 5 or REG3A protein expression. Another antimicrobial peptide, e.g., lysozyme, might mediate the antibacterial effect of C12-O-AHL. In mouse nasal epithelial cells, the quorum-sensing molecules *N*-butyryl-l-homoserine lactone and C12-O-AHL stimulated calcium-dependent NO production that increased mucocilliary clearance (33). This mechanism required components of the taste signaling pathway and suggested that TAS2Rs are involved in innate immune responses induced by quorum-sensing molecules.

Surprisingly, the natural bitter TAS2R43 agonists, quinine and aloin, stimulated the release of molecules from epithelial cells of TAS2R43^+^ patients that enhanced bacterial growth. The effects on *E*. *coli* growth were most pronounced with supernatants of aloin-stimulated crypts from patients with the TAS2R43^+^ W genotype, who, according to oral taste tests, have a higher sensitivity to aloin than do individuals with the TAS2R43^+^ S genotype. Therefore, we assume that the sensitivity of perception of aloin in the gut depends on the TAS2R43 polymorphism in a manner similar to that seen in the lingual system.

Proteomics analysis revealed that aloin treatment of jejunal crypts from TAS2R43^+^ patients induced the secretion of CLCA1, a glycoprotein stored in secretory vesicles of goblet cells that cleaves mucin 2 in the mucus structural transition zone (26). CLCA1, therefore, takes part in mucus processing and in the structural arrangement of the mucus (26). In addition, CLCA1 participates in innate immune responses by altering cytokine and chemokine production (25). In our study, mucin 2 and CLCA1 protein release were correlated, indicating that these 2 glycoproteins could be responsible for the detected enhancement of *E*. *coli* growth in the CFU assay. Cleaved carbohydrates from glycoproteins could be a nutritional source for microorganisms (37). Even though *E*. *coli* is not one of the major mucin-degrading bacteria in the gut, it produces β-galactosidase that enables the cleavage of carbohydrates (38).

Analysis of mRNA expression via qPCR in jejunal crypt cultures after 4 hours of treatment with DB revealed a decrease in α-defensins expression. The bulk RNA-Seq analysis of the same RNA samples confirmed the decrease in the expression of the antimicrobial peptides (*DEFA5*, *DEFA6*, *REG3A*) and mucus proteins (*AGR2*, *CLCA1*, *ZG16*), but showed an increase in the expression of *MUC17*. MUC17 is a long transmembrane mucin produced by goblet cells that can be induced by carbachol or TNF-α (39), maintains the epithelial barrier of the crypts, and protects the epithelial cells from bacterial invasion (39, 40). The decrease in mRNA expression of antimicrobial peptides after 4 hours of incubation of primary crypt cultures with DB was in contradiction with the acute release of α-defensin 5 and REG3A after 30 minutes of stimulation. Prolonged exposure to DB was shown to activate other signaling pathways like the NRF2-mediated oxidative stress response and the unfolded protein response. The discordance between our findings might be a consequence of a translational block caused by the stress response induced by DB to maintain homeostasis.

DB decreased expression of the chemokines *CCL2*, *CCL3*, and *CCL4*. Pathway analysis revealed that these chemokines are involved in differential regulation of cytokine production in intestinal epithelial cells by IL-17A and IL-17F. The release of IL-17 (IL-25 in mice) can be initiated by activation of bitter taste receptors in intestinal tuft cells via bitter compounds (41) or secretory products of parasitic helminths (42, 43). This indicates that in our preparation other cell types might also have been activated by DB and were able to induce a type 2 immunity response besides the alteration of α-defensin expression.

We show, for the first time to our knowledge, that DB strongly activated the NRF2-mediated oxidative stress response and the unfolded protein response. Both pathways are a central part of the mitohormetic response that controls aging processes and metabolic stress. A link between these pathways and the transcription factor *ATF4* regulating the transcription of the mitokines GDF15 and ADM2 has been described previously (44). These genes were also upregulated by DB treatment in jejunal crypts in our study. These genes were also upregulated by DB treatment in jejunal crypts. The increase in *GDF15* was independent of TAS2R43. Previous studies already showed that the bitter-tasting compounds diclofenac (TAS2R14) and resveratrol (TAS2R14, TAS2R39) increased the NSAID-activated gene (NAG-1), which was later renamed GDF15, in a human colorectal carcinoma cell line (45, 46). GDF15 triggers conditioned taste aversion in response to nutritional stress and has anorectic and weight-suppressive effects (28, 47, 48). It was recently shown that the weight loss associated with metformin treatment is mediated by an increase in circulating GDF15 levels (49, 50). Antibodies against the receptor for GDF15 reverses cancer cachexia in mice (47). Furthermore, GDF15 is also released by the cancer therapeutic drug cisplatin to mediate anorectic signaling (51). The increased expression of GDF15 could be a mechanism of the human body to manifest an aversion to bitter compounds also when it is “sensed” in the gut. In addition, an increase in GDF15 expression could explain the reduced hunger scores after intragastric administration of DB to healthy volunteers and the reduction in food intake induced by administration of bitter compounds to mice and humans (5, 9, 52, 53). The FC in *GDF15* expression after treatment with DB correlated with the FCs in expression of *ADM2* and the *LDLR*, indicating that DB treatment induced mRNA expression of genes that are involved in food intake and triglyceride metabolism. In fact, studies in diet-induced obese mice and prediabetic obese humans have shown that the bitter compound KDT501 improved triglyceride levels (54, 55).

Transcriptomics analysis revealed that aloin enhanced the translation of proteins and induced oxidative phosphorylation probably to produce more energy for the transport of vesicles that contained, for example, glycoproteins in crypts from TAS2R43^+^ patients. This hypothesis is supported by the increase in *ANXA2* mRNA expression detected in our RNA-Seq analysis. ANXA2 is a Ca^2+^ regulated membrane-binding protein that functions in secretory vesicle exocytosis by providing membrane bridges (30). The release of the glycoprotein CLCA1, which is transported via vesicles to the cell membrane, was confirmed by proteome analysis after aloin treatment.

A limitation of our findings is that we conducted the TAS2R43^+^ versus TAS2R43^–^ studies solely in jejunal crypts derived from patients with obesity. There was a lower availability of the organ donor tissue, therefore, we were unable to collect enough tissue from TAS2R43^–^ lean individuals. Thus, the described effects elicited by TAS2R43 via aloin are limited to obese individuals. Further, in the RNA-Seq analysis, we found fewer or no DEGs after DB or aloin treatment of crypts from lean individuals. Because of technical issues, this analysis could only be performed in 4 patients, which could explain the lower number of identified DEGs. Nevertheless, the DEGs identified after DB treatment overlapped with those from the obese data set and also included *GDF15* and *CCL2*. This suggests that, even though the effects induced by DB were less pronounced in crypts from lean subjects, similar mRNA expression markers were affected that are involved in the regulation of immune responses and body weight control.

In conclusion, this is the first study to our knowledge to show that bitter compounds can activate several pathways in intestinal crypts, especially those derived from individuals with obesity, to elicit defense mechanisms that affect *E*. *coli* growth. The broadly tuned bitter compound DB altered the mRNA expression of several antimicrobial peptides, mucus proteins, and cytokines (CCL2-4) that are part of the type 2 innate immune response. We identified TAS2R10 as a potential target to alter antimicrobial peptide release in the human intestine. Furthermore, aloin induced CLCA1 release, activated oxidative phosphorylation, and affected mRNA expression of genes involved in vesicle transport and protein translation in a TAS2R43-dependent manner. CLCA1 plays an essential role in goblet cell mucus production and innate immunity of respiratory and gastrointestinal diseases such as cystic fibrosis, ulcerative colitis, and gastrointestinal parasitic infection (25). Therefore, targeting TAS2R43 to treat these diseases might be of interest. Defining taste receptor polymorphisms in patients could assist in optimizing therapies for personalized treatments. Furthermore, DB induced an NRF2-mediated stress response and an unfolded protein response that resulted in increased expression of the mitokines *GDF15* and *ADM2* and the *LDLR* genes, which control anorectic signaling and lipoprotein homeostasis. A potential application in which bitter compounds can induce a mild nutritional stress response that may be beneficial in body weight control needs further investigation.

## Methods

### Chemicals.

DB, SB, PTC, PHE, FFA, quinine, aloin, TA, PGG, BERB, ARG, CuE, C8-AHL, and C12-O-AHL were purchased from MilliporeSigma.

### Study groups.

Full-thickness small intestinal tissue (jejunum) was obtained either from lean (BMI <25, nondiabetic) multiorgan donors (donation after circulatory death [DCD], 56%; or donation after brain death [DBD], 44%) or from patients with obesity (BMI >30, nondiabetic) who underwent Roux-en-Y gastric bypass (RYGB) surgery. Donors older than 50 years of age are not considered suitable for intestinal transplantation. If tissue from a donor under 50 years of age was used, it was because no suitable recipient was found for the intestine. The pre-procurement pancreas suitability score used by Eurotransplant for the donor tissues used in this study was higher than 17, and thus neither the pancreas nor the intestine was deemed suitable for transplantation. Five of the 9 donors had experienced a craniocerebral trauma. The agonal phase of the DCD III donors (*n =* 5) ranged from 12–27 minutes (average 19 ± 3 min). The donors still had cardiac output and a mean arterial pressure of 50 mmHg. The time between cardiac arrest and cold perfusion for these donors was limited to 8 minutes. Five of 9 organ donors received noradrenalin in concentrations ranging from 0.05–0.30 μg/kg/min (average 0.16 ± 0.04 μg/kg/min). The potassium levels ranged from 3–5.5 mmol/L (average 3.96 ± 0.25 mmol/L), and the sodium levels ranged from 133–149 mmol/L (average 143 ± 2 mmol/L). Detailed protocols for human tissue procurement have been described previously (56). Patient and donor demographics are shown in Supplemental Table 1.

### Primary culturing of isolated human jejunal crypts.

A piece of mucosal layer was dissected from the fresh jejunal tissue, minced, and digested with collagenase XI (0.35 mg/mL) in DMEM at 37°C. Crypt suspensions were centrifuged, resuspended in DMEM (supplemented with 10% FBS, 1% penicillin and streptomycin, 1% l-glutamine, and 10 μM Y-27632), and then filtered (100 μM). Crypts were seeded onto Matrigel-coated (1.4% (v/v) 12-well plates or glass coverslips and incubated for 24 hours at 37°C to form a monolayer.

### Immunofluorescence studies.

Primary jejunal crypts were fixed with 4% (w/v) paraformaldehyde for 30 minutes. Jejunal tissue was fixed (2 h) and cryoprotected overnight in 30% sucrose at 4°C and frozen. Primary crypts and tissue sections (10 μm) underwent antigen retrieval (10 mM sodium citrate solution, pH 6, for 30 min at 80°C), followed by preincubation with PBS containing 10% (v/v) donkey serum and 0.3% (v/v) Triton X-100 for 2 hours. Samples were incubated overnight at 4°C with the following primary antibodies: mouse anti–α-defensin 5 (1:200; ab90802, Abcam); rabbit anti–α-defensin 6 (1:200; 17923-1-AP, Proteintech); rabbit anti-lysozyme (1:200; A0099, Dako); mouse anti–mucin 2 (1:200; MA5-12345, Thermo Fisher Scientific); rabbit anti-TAS2R10 (1:100; PA5-39708, Thermo Fisher Scientific); and/or rabbit anti-TAS2R43 (1:100; PA5-103257, Thermo Fisher Scientific). Samples were washed and incubated with donkey anti–rabbit Cy5 (1:800; 711-175-152, Jackson ImmunoResearch) or donkey anti–mouse Cy3 (1:800; 715-165-150, Jackson ImmunoResearch) secondary antibody for 2 hours. Nuclei were stained with DAPI (D1306, Thermo Fisher Scientific). For the negative control, primary antibodies were replaced with normal rabbit serum. Coverslips were mounted with mounting media AF1 (Citifluor, Electron Microscopy Sciences) and imaged using an Olympus BX41 fluorescence microscope. For the colocalization studies, at least 3 views per slide from at least 3 patients were analyzed. For the quantification studies, images were taken at the same exposure time, and the median fluorescence intensity of stained cells was quantified in at least 5 different views from 1 culture preparation from each patient (*n* = at least 4 patients) using ImageJ (NIH).

### Single-cell calcium imaging.

Primary jejunal crypts were loaded for 30 minutes with Fluo-4 AM (5 μM) in HEPES buffer and transferred to a coverglass chamber mounted on a Nikon Eclipse TE300 inverted fluorescence microscope. Cells were stimulated via a local perfusion system (1 mL/min) with DB (0.1–1 mM) or PTC (0.1 mM) for 60 seconds, and changes in intracellular Ca^2+^ concentrations were recorded with TILLvisION software on a Nikon TE microscope equipped with a PolyV monochromator (TILL Photonics) and Sensicam-QE CCD camera (PCO). Image analysis was performed with custom-written routines in Igor Pro (Wavemetrics) (57) and macros in Excel (Microsoft). Regions of interest were drawn, the background was subtracted, and the fluorescence intensity was normalized to the basal fluorescence at the onset of the recording (first 50 s) for each region of interest (ΔF/F_0_). Only signals that rose above baseline plus 2 times the intrinsic noise level were considered. After Ca^2+^ imaging, cell preparations were fixed for 30 minutes with 4% paraformaldehyde, and immunofluorescence staining for α-defensin 5 and mucin 2 was performed as described above. The images were adjusted for contrast and brightness.

### Western blot analysis.

Primary crypts were stimulated for 30 minutes with Krebs containing vehicle or bitter compounds. The supernatant was removed, and cells from 3 wells were lysed with lysis buffer (50 mM Tris-HCl, 150 mM NaCl, 0.5% w/v sodium deoxycholate monohydrate, 1% v/v IGEPAL CA-630 and EDTA-free protease inhibitor cocktail), pooled, centrifuged, and stored at –80°C.

Samples (20 g) were separated by SDS-PAGE (NuPAGE 4%–12% Bis-Tris, 1.0 mm) and transferred onto Invitrolon polyvinylidene difluoride membranes (Thermo Fisher Scientific). Blots were incubated overnight with the primary antibodies rabbit anti–α defensin 5 (1:400, PA5-82540, Thermo Fisher Scientific) or rabbit anti-REG3A (1:300; PA5-23341, Thermo Fisher Scientific). All membranes were stained with mouse anti–vinculin (1:10,000; V9264, MilliporeSigma) as a protein loading control. Peroxidase-conjugated goat anti–rabbit IgG (1:5000; 32460, Thermo Fisher Scientific) or goat anti–mouse IgG (1:5000; 32430, Thermo Fisher Scientific) was used as a secondary antibody. The membranes were treated with SuperSignal West Femto Maximum Sensitivity Substrate (34095, Thermo Fisher Scientific) and imaged using the ImageQuant LAS 4000 Mini (GE Healthcare). Bands were quantified using ImageJ software. A calibrator prepared from nonstimulated crypts was run on every Western blot to correct for inter-run variability. The chemiluminescent signal intensity was corrected for the calibrator and expressed relative to the protein-loading control vinculin. The effect of the bitter compound was expressed as a percentage of the vehicle stimulation in the crypt preparation from the same patient.

### Antibacterial activity.

Primary jejunal crypts were incubated with a cholinergic agonist (10 μM carbachol); bitter agonists (0.1 mM–1 mM DB, 1 mM SB, 1 mM PTC, 0.1 and 1 mM PHE, 30 μM FFA, 0.1 mM quinine, 30 μM aloin, 10 μM PGG, 0.5 mM TA, 10 μM BERB, 10 μM ARG, 0.1 μM CuE, 100 μM C8-AHL, 25 μM C12-O-AHL); or vehicle in Krebs-Ringer buffer for 30 minutes at 37°C (see Table 1). Concentrations were determined on the basis of effective concentrations for receptor stimulation evaluated by Ca^2+^ measurements in a heterologous expression model of TAS2Rs in human embryonic kidney (HEK) cells (20–23). The cytotoxic effects of these compounds at the tested concentrations on jejunal crypts were excluded by a neutral red uptake assay (58), and cell viability was higher than 85%. The CSNs were collected for determination of their effect on the number of viable *E*. *coli* CFU.

### Quantification of CFU.

*E*. *coli* (American Type Culture Collection [ATCC], 25922) were grown overnight in 50 mL BD tryptic soy broth medium (Thermo Fisher Scientific) at 37°C with shaking. The suspension was diluted (1:100) in 20 mL fresh broth medium and subcultured for 3 hours to attain a mid-log-phase culture (OD at 600 nm = 0.6–1.0). Bacteria were centrifuged, washed, resuspended in the supernatant of crypts treated with bitter compounds (5 × 10^5^ cells/mL), and incubated for 4 hours at 37°C with shaking. The bacterial suspensions were further diluted and spread on agar plates. After overnight incubation, the formed colonies were counted.

### Proteome analysis of CSN by liquid chromatography–tandem mass spectrometry.

The CSN samples were also subjected to proteomics analysis. Proteins were precipitated by adding 4 volumes of 96% EtOH at –20°C overnight and dried. S-trap technology was applied for proteomics sample processing as described previously (59). Eluted peptides were dried and dissolved in 5 μL 30% formic acid, supplemented with 4 synthetic peptide standards for internal quality control, and further diluted with 40 μL mobile phase A (97.9% H_2_O, 2% acetonitrile, 0.1% formic acid) (60). Five microliters was injected into a Dionex UltiMate 3000 RSLC nano LC system coupled to a Q Exactive Orbitrap MS (Thermo Fisher Scientific). Peptides were trapped on a C18 2 cm/100 μm column, and LC separation was performed on a 50 cm × 75 μm PepMap 100 analytical column (Thermo Fisher Scientific). A gradient from 7% to 40% mobile phase B (79.9% acetonitrile, 20% H_2_O, 0.1% formic acid) at a flow rate of 300 nL/min within 43 minutes was applied, with a total run time of 85 minutes including washing and reequilibration. Mass resolution on the MS1 level was set to 70,000 (at *m/z* = 200) with a scan range from 400 to 1400 *m/z*. The top 8 most-abundant peptide ions were chosen for fragmentation at 30% normalized collision energy, and the resulting fragments were analyzed in the Orbitrap at a resolution of 17,500 (at *m/z* = 200). MaxQuant (version 1.6.14.0) was used for protein identification at previously described settings (61), and LFQ values were determined. Biostatistical analysis was performed on the Perseus software platform, version 1.6.6.0 (62). A paired, 2-sample Student’s *t* test was used to determine secreted proteins that were significantly different between the supernatants from aloin- and Krebs-treated (control) crypts, with a permutation-based FDR of 0.05, S0 = 0.

### RNA isolation for qPCR and RNA-Seq.

Jejunal primary crypts were stimulated with DMEM (control) or bitter agonists (0.1–1 mM DB or 30 μM aloin) in DMEM for 4 hours at 37°C. The supernatant was removed, and total RNA was isolated from cells (Relia Prep Kit, Promega) and frozen at –80°C.

### qPCR.

Total RNA was reverse transcribed to cDNA using the High Capacity cDNA Reverse Transcription Kit (Thermo Fisher Scientific). qPCR was performed using the LightCycler 480 with LightCycler 480 SBYR Green I Master Mix (Roche Diagnostics). The primers used are listed in Supplemental Table 2. The primers for TAS2Rs were designed and validated previously (7, 63). Amplicons for each gene were extracted from agarose gel electrophoresis with the QiaQuick Gel Extraction Kit (QIAGEN) and sequenced (LGC Genomics) to confirm primer specificity. Raw data were analyzed using the software LinReg PCR to determine PCR efficiency. Cq values were normalized to a calibrator to correct for inter-run variations and to the geometric mean of the normalized expression of the stable endogenous control genes (*GAPDH*), ribosomal protein S18 (*RPS18*), and ribosomal protein S11 (*RPS11*) (efficiency^–ΔΔCq^ method).

### Genotyping TAS2R43.

The TAS2R43 deletion polymorphism was determined by qPCR on the cDNA samples prepared as described above. The SNP rs68157013 from TAS2R43 was determined as described previously (18). PCR products were Sanger sequenced at LGC Genomics and analyzed with the freeware Chromas, version 2.6.2 (www.technelysium.com.au). Identification of the nucleotide G at position 104 should translate to W and was considered the TAS2R43^+^ W genotype (highly sensitive to aloin). Identification of a C at this position should translate to S and was considered the TAS2R43^+^ S genotype (less sensitive to aloin).

### RNA-Seq.

Bulk RNA-Seq was performed by the Genomics Core Leuven. In brief, sequence libraries were prepared with the Lexogen QuantSeq 3′ mRNA-Seq Library Prep Kit (Lexogen), and quality and size range were assessed using the Bioanalyzer with the DNA 1000 kit (Agilent Technologies). Libraries (2 nM) were sequenced using an Illumina HiSeq 4000 platform (single-end reads of 50 bp length with a minimum of 1 × 10^6^ reads per sample). Quality control of raw reads was analyzed with FastQC, version 0.11.7 (64), and adapters were filtered with ea-utils fastq-mcf, version 1.05 (65). Splice-aware alignment was conducted with STAR, version 2 (66), against the human reference genome hg19 using the default parameters. Reads that mapped to multiple loci in the reference genome were discarded. The resulting BAM alignment files were processed with Samtools, version 1.5 (67). The quantification of reads per gene was calculated with HT-Seq Count, version 2.7.14. Count-based differential expression analysis on paired samples was performed with the R-based Bioconductor package DESeq2 (68). Reported *P* values were adjusted for multiple testing with the Benjamini-Hochberg procedure, which controls the FDR, indicated as the *q* value. Functional analyses and the identification of canonical pathways and upstream regulators were performed using Ingenuity Pathway Analysis (IPA) software (QIAGEN). Correlation analysis after Pearson’s correlation coefficient analysis of the 120 identified significantly DEGs was calculated using GraphPad Prism 8. Heatmaps and hierarchical clustering (minus one Pearson) were generated using the freeware tool Morpheus (https://software.broadinstitute.org/morpheus). The RNA-Seq data were deposited in the NCBI’s Gene Expression Omnibus (GEO) database (69) (GEO GSE186509; https://www.ncbi.nlm.nih.gov/geo/query/acc.cgi?acc=GSE186509).

### Statistics.

Results are presented as the mean ± SEM. Data were assessed for normality of distribution in SAS (SAS/Stat 14.1, SAS Institute). Non-normally and/or nonhomogeneously distributed data underwent log transformation for statistical analysis. Differences in age and BMI between lean and obese individuals were analyzed using an unpaired, 2-tailed Student’s *t* test. Sex difference between populations was analyzed using the χ^2^ calculation in GraphPad Prism 9.2.0 (GraphPad Software). Western blot analysis was performed using a paired, 1-tailed Student’s *t* test followed by Bonferroni-Holm correction for multiple comparisons. All other statistical analyses were performed using the mixed model analysis in SAS (SAS/Stat 14.1). Differences in mRNA expression and fluorescence intensity of the immunostainings were analyzed using a mixed model with patient as the random effect to determine both the between-subjects and within-subjects effects. For the CFU assay, both the patient and experimental day were considered random effects and analyzed using a mixed model. The effect of the different bitter compounds at the crypt-dependent and -independent levels as well as the influence of the patient’s TAS2R43 genotype were considered as well. In the qPCR analysis, for each bitter compound tested on the crypts and per gene analyzed, the influence of obesity and the TAS2R43 genotype on the bitter compound effect was determined. Post hoc tests with Šidák’s correction for multiple testing were applied. A *P* value of less than 0.05 was considered significant.

### Study approval.

Written informed consent was obtained from all participants with obesity recruited at the University Hospital UZ Leuven (Leuven, Belgium) for this study. According to Belgian laws, every citizen is a potential donor, and no specific consent is needed for organ or tissue donation. In cases of donation, tissue can be used for scientific research if the local ethics committee agrees. Ethics approval for the study was provided by the Medical Ethics Committee UZ/KU Leuven (S56978 [organ donors] and S57826 [patients with obesity]) and performed in accordance with Declaration of Helsinki principles.

## Author contributions

KIL, ID, and QW conceived and designed all the experiments. AS contributed to the design of the bacterial assays. ED, BVDS, LJC, and ML recruited the patients. KIL, QW, TT, LN, and MF performed the experiments. CG and BN performed the liquid chromatography–tandem mass spectrometric analysis and processed proteomics data. JT provided functional set-ups. KIL interpreted the transcriptomic and proteomic data. KIL, QW, and ID analyzed the results. TT performed the statistical analysis. KIL, ID, and QW wrote the manuscript. All authors reviewed the manuscript.

## Supplementary Material

Supplemental data

## Figures and Tables

**Table 1:**
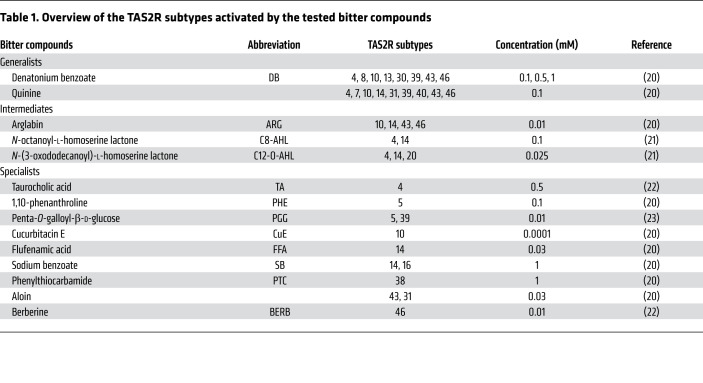
Overview of the TAS2R subtypes activated by the tested bitter compounds

## Version 1 11/16/2021

In-Press Preview

## Version 2 02/01/2022

Electronic publication
