# Supplementary material for: Human intestinal bitter taste receptors regulate innate immune responses and metabolic regulators in obesity
Source: J Clin Invest. 2022 Feb 1;132(3):e144828. doi: 10.1172/JCI144828 (PMC8803326; doi:10.1172/JCI144828)
Supplement: Supplemental data [file jci-132-144828-s166.pdf]

Supplemental Tables

Supplemental Table 1. Demographics of lean and obese subjects

Parameter	Lean (n=9)	Obese (n=42)	Lean vs Obese
**Male/Female**	7/2	8/34	P<0.001
**Age (years)**	57±3	47±2	P<0.05
**BMI (kg/m^2)**	23.9±1.1	40.8±0.7	P<0.001

Supplemental Table 2. Primers used in qPCR

**Genes**	**Forward primer**	**Reverse primer**	**Size (bp)**
TAS2R4	GCAGTGTCTGGTTTGTGACC	GCGTGATGTACAGGCAAGTG	168
TAS2R5	ACACTCATGGCAGCCTATCC	CGAGCACACACTGTCTTCCA	107
TAS2R7	GCAGGTGTGGATGTCAAACTC	TCTTGACCCAGTCCATGCAG	167
TAS2R8	ATGTGGATTACCACCTGCCT	GGAAATGGCAAAGCATCCCAG	135
TAS2R10	CCTTTGGAGACACAACAGGC	GACCCCAGGGATAGATGGCT	221
TAS2R13	GAAAGTGCCCTGCCGAGTAT	CCAGATCAGCCCAATTCTGGA	177
TAS2R14	CCAGGTGATGGGAATGGCTTA	AGGGCTCCCCATCTTTGAAC	128
TAS2R16	ATGGCATCACTGACCAAGCA	TTTCAACGTAGGGCTGCTCA	255
TAS2R20	ATTTGGGGGAACAAGACGCT	ACTACGGAAAAACTTGTGGGAA	183
TAS2R30	GGCTGGAAAAGCAACCTGTC	ACACAATGCCCCTCTTGTGA	191
TAS2R31	TTGAGGAGTGCAGTGTACCTTTC	ACGGCACATAACAAGAGGAAAA	218
TAS2R38	AGGCCCACATTAAAGCCCTC	CAGCTCTCCTCAACTTGGCA	216
TAS2R39	TTCTGTGGCTGTCCGTGTTTA	GGGTGGCTGTCAGGATGAAC	207
TAS2R40	CGGTGAACACAGATGCCACAGATA	GTGTTTTGCCCCTGGCCCACT	150
TAS2R43	ATATCTGGGCAGTGATCAACC	CCCAACAACATCACCAGAATGAC	148
TAS2R46	ACATGACTTGGAAGATCAAACTGAG	AGCTTTTATGTGGACCTTCATGC	200
RPS11	CAGCCGACCATCTTTCAAAAC	TCTCGAAGCGGTTGTACTTG	274
RPS18	ACCAACATCGATGGGCGGCG	TGGTGATCACACGTTCCACCTCA	157
GAPDH	AGGTGAAGGTCGGAGTCA	GGTCATTGATGGCAACAA	99
DEFA5	GCCATCCTTGCTGCCATTC	TGATTTCACACACCCCGGAGA	241
DEFA6	CCTCACCATCCTCACTGCTGTTC	CCATGACAGTGCAGGTCCCATA	266
DEFB1	ATGAGAACTTCCTACCTTCTG	TCACTTGCAGCACTTGGCC	207
DEFB4A	CCAGCC ATCAGCCATGAGGGT	GGAGCCCTTTCTGAAT CCGCA	255
MUC1	CATTGCCTTGGCTGTCTGTC	GCGACGTGCCCCTACAAG	246
MUC2	CAGCACCGATTGCTGAGTTG	GCTGGTCATCTCAATGGCAG	140
TAS2R43-Sequencing	CCAGTCTGGTAGTGGTTACA	TCACCCAGTACCTCATTTGCC	876
GDF-15	TCCAGACCTATGATGACTTGT	AACCTTGAGCCCATTCCA	127

Supplemental Figures

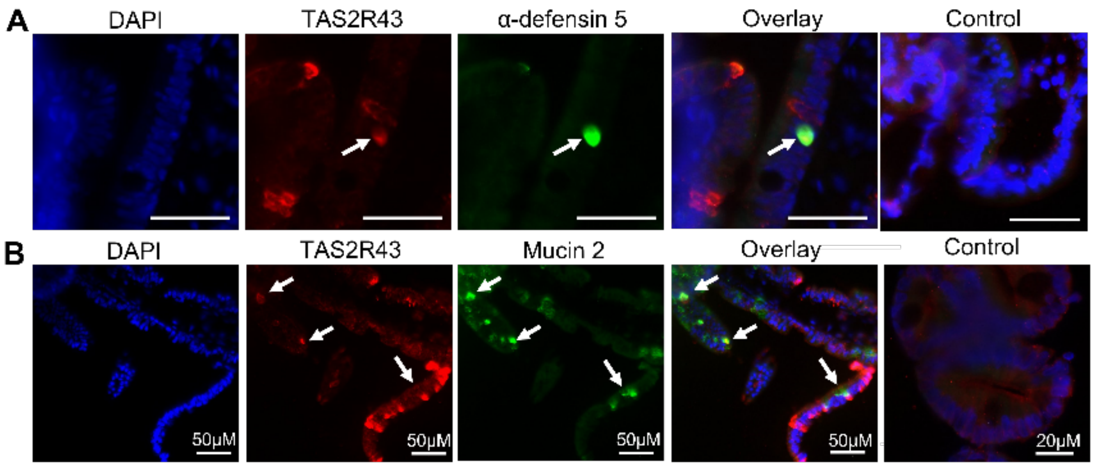

Supplemental Figure 1. Co-localization of TAS2R43 with Paneth or goblet cells in tissue sections from a lean subject. Representative double-immunofluorescence staining of jejunal tissue sections from a lean organ donor for TAS2R43 (red) and (**A**) α-defensin 5, Paneth cells, or (**B**) mucin 2, goblet cells, (green). Nuclei were stained by DAPI (blue). Scale bars, (**A**) 50μm or (**B**) as indicated. Co-localization was performed in sections from two lean subjects.

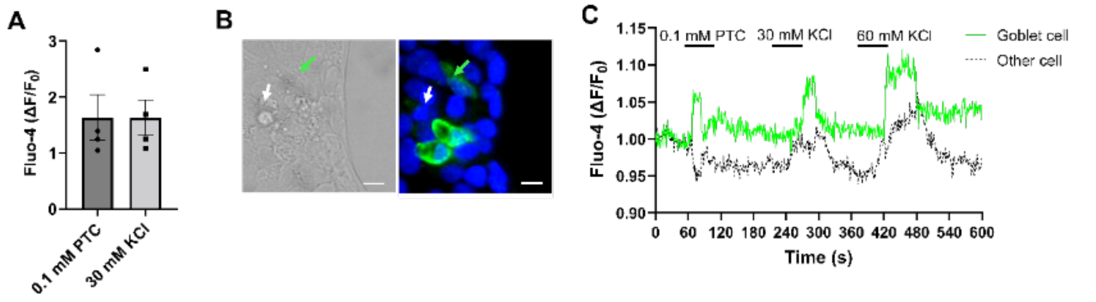

Supplemental Figure 2. Intracellular Ca^{2+} changes in response to PTC and identification of cells in crypts from obese patients by immunostaining. (**A**) Relative rise in fluorescence intensity in single cells from primary jejunal crypts from obese patients treated with 0.1 mM PTC or 30 mM KCl. Data represent the mean±SEM, n=4 obese subjects. (**B**) The left picture shows the cells in brightfield of which the tracings of the Ca^{2+} changes are depicted. The right picture shows the immunofluorescent staining for mucin 2, a marker for goblet cells. (**C**) Tracing of a goblet cell responding to 0.1 mM PTC with a Ca^{2+} increase and a non-identified, non-responding cell. Scale bars=20 µM.

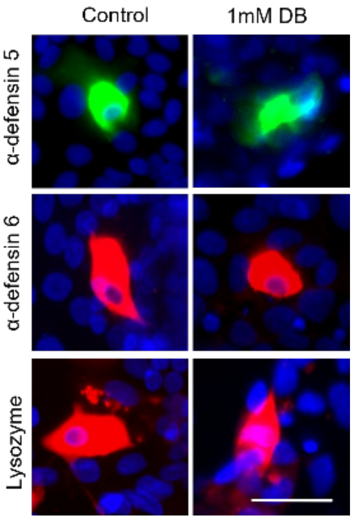

Supplemental Figure 3. Immunofluorescence study of the effect of DB on protein expression in Paneth cells in a lean subject. Representative immunofluorescence staining for α-defensin 5, α-defensin 6 and lysozyme after 30 min treatment of jejunal crypts from a lean patient with DMEM or 1mM DB. No difference between control and 1 mM DB treatment was detected. Scale bar, 25µm.

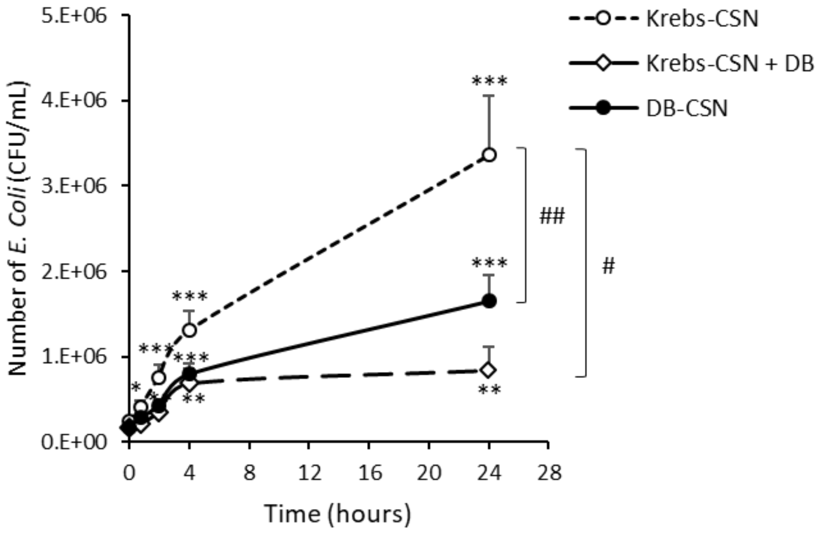

Supplemental Figure 4. **Bacteriostatic effects of DB on *E. coli* growth.** Time-dependent effect of the supernatant of jejunal crypts from obese patients stimulated for 4 hours with Krebs (Krebs-CSN) or DB (DB-CSN) on colony forming units (CFU) of *E. coli* and their bacteriostatic control (Krebs-CSN + DB). Data represent the mean±SEM, (n=4-6). Statistics: mixed model over time *: $P<0.05$, **: $P<0.01$, ***: $P<0.001$, vs. 0h; #: $P<0.05$, ##: $P<0.01$, vs. Krebs-CSN.

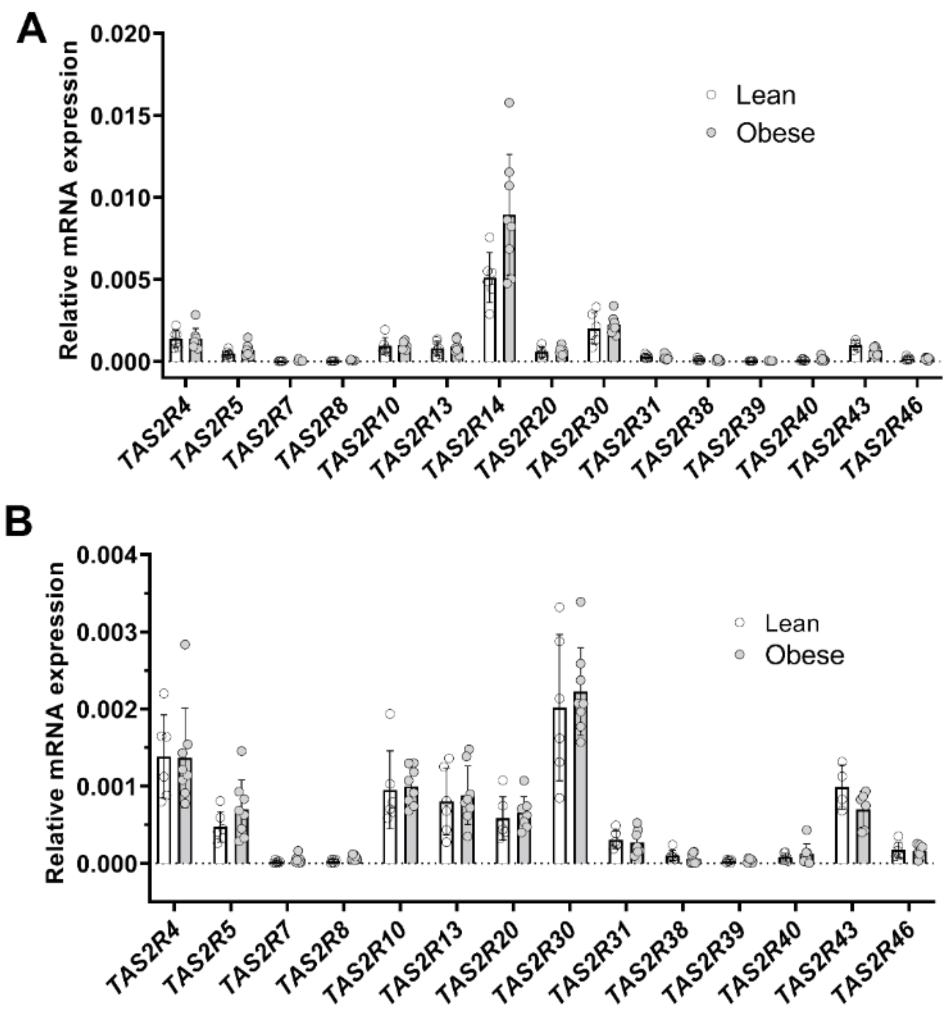

Supplemental Figure 5. Relative mRNA expression of TAS2Rs in crypts derived from either lean or obese subjects. (**A** and **B**) Relative mRNA expression of single TAS2Rs in primary jejunal crypts from lean (n=4-6) and obese (n=6-8) subjects normalized to the expression of the endogenous controls RPS18, GAPDH and RPS11. (**B**) Detail of (**A**) excluding TAS2R14. Data represent the mean±SEM. Statistics: Mixed model lean vs. obese. No significant differences between the expression of TAS2Rs in lean vs. obese was detected.

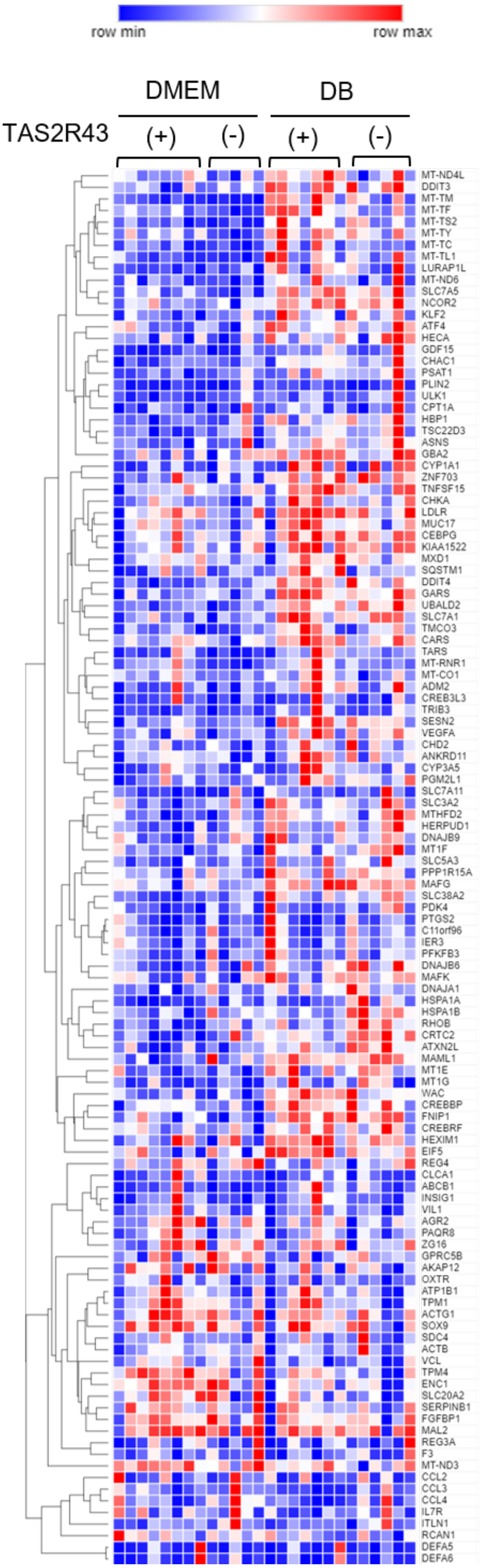

Supplemental Figure 6. Heatmap of the 120 identified DEGs identified in jejunal crypts from obese TAS2R43(+) and TAS2R43(-) patients after 4 hours treatment with DMEM or 1mM DB. Hierarchical clustering was conducted by one minus Pearson correlation, linkage method average, with the heatmapping tool Morpheus of the Broad Institute.

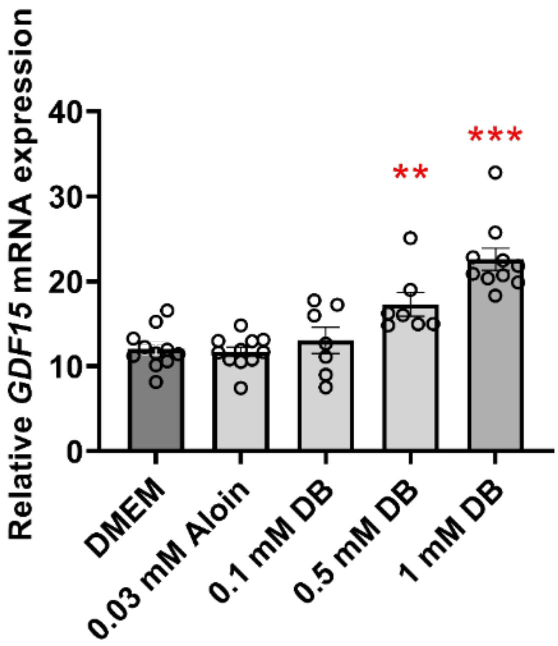

Supplemental Figure 7. Effect of bitter agonists on *GDF15* mRNA expression in crypts from obese patients. Concentration-dependent effect of treatment of jejunal crypts from obese patients (n=7-11) for 4 hours with DB (0.1-1 mM) on relative *GDF15* mRNA expression. Treatment with 0.03 mM aloin for 4h had no effect on *GDF15* mRNA expression. Data represent the mean±SEM. Statistics: mixed model. **: P< 0.01, ***: P < 0.001, vs. DMEM.

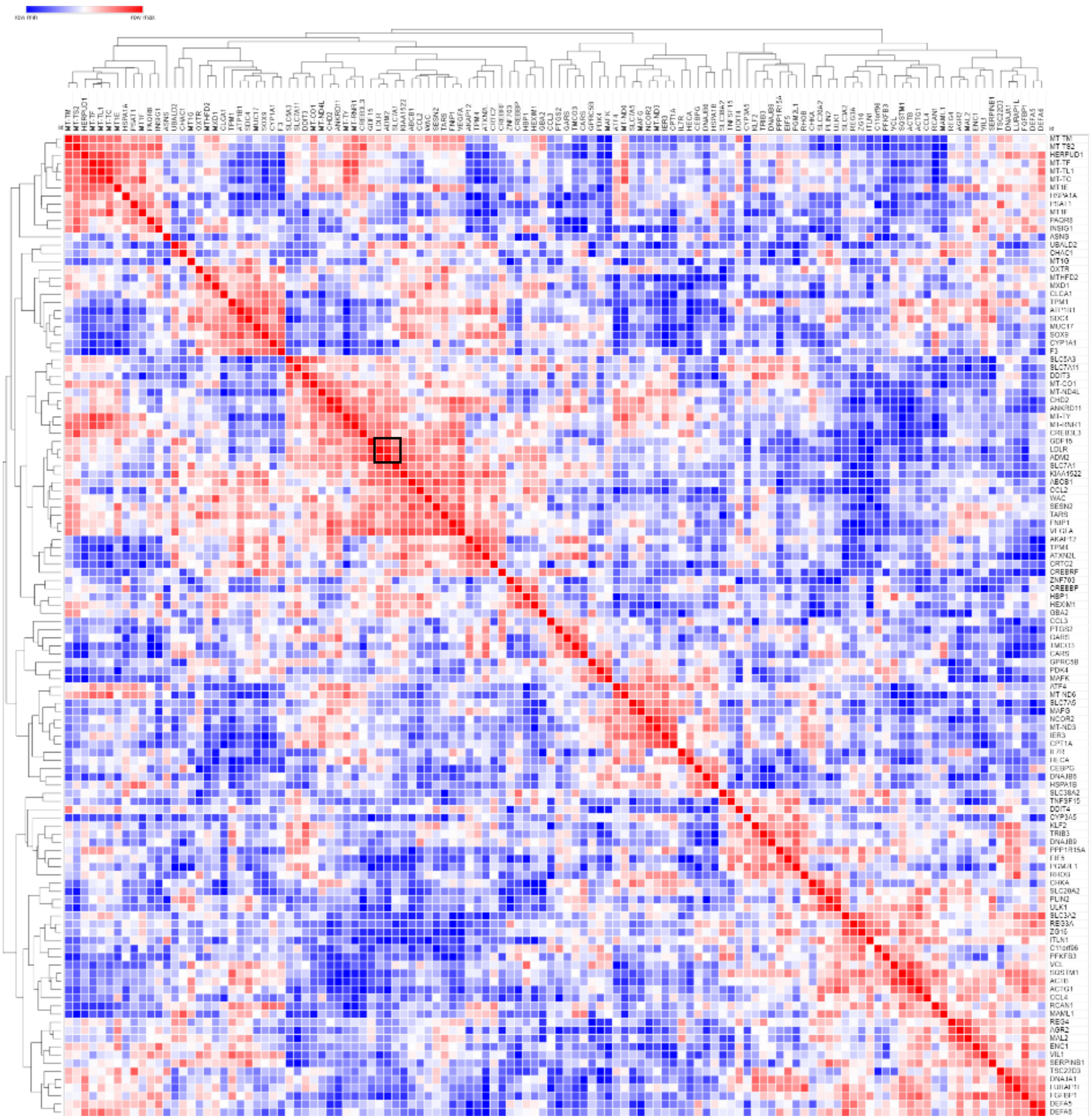

Supplemental Figure 8. **Correlation matrix of the fold changes of 120 identified DEGs in response to DB in jejunal crypts from obese patients.** The most significantly upregulated gene *GDF15* significantly correlated with *ADM2* and *LDLR*, position indicated by a black square (Pearson r).

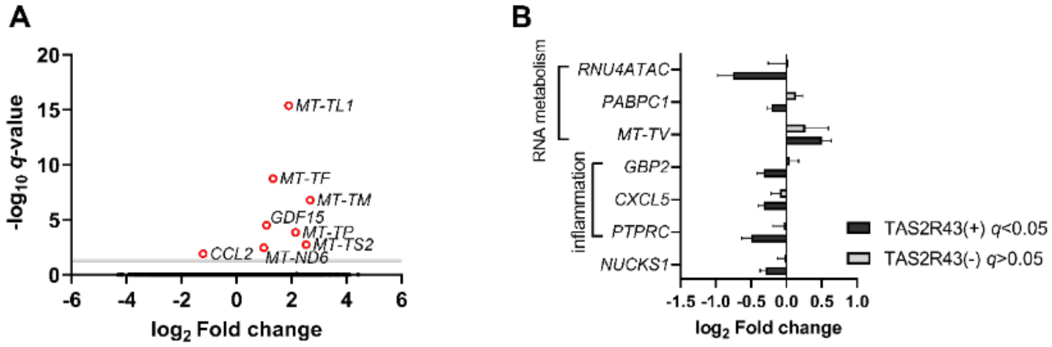

Supplemental Figure 9. **Transcriptomic analysis of jejunal crypts from lean or obese subjects stimulated with DB.** (**A**) Volcano plot showing the log_2 fold changes (DB-DMEM) of significantly DEGs indicated in red, after treatment of crypts derived from lean subjects (n=4) with 1 mM DB or DMEM (control). (**B**) Comparison of the log_2 fold changes of the 7 DEGs identified only in the data set of the TAS2R43(+) obese subjects (n=7-8) after treatment with 1 mM DB vs TAS2R43(-) (n=5-6).
